# Reliability of detection of ultrasound and MRI features of hand osteoarthritis: a systematic review and meta-analysis

**DOI:** 10.1093/rheumatology/keab470

**Published:** 2021-06-04

**Authors:** Abasiama D Obotiba, Subhashisa Swain, Jaspreet Kaur, Michael Doherty, Weiya Zhang, Abhishek Abhishek

**Affiliations:** Academic Rheumatology, School of Medicine, University of Nottingham, Nottingham; Department of Medical Imaging, College of Medicine and Health, University of Exeter, Exeter; Academic Rheumatology, School of Medicine, University of Nottingham, Nottingham; Department of Primary Care, University of Oxford; Academic Rheumatology, School of Medicine, University of Nottingham, Nottingham; Academic Rheumatology, School of Medicine, University of Nottingham, Nottingham; Academic Rheumatology, School of Medicine, University of Nottingham, Nottingham; Academic Rheumatology, School of Medicine, University of Nottingham, Nottingham; NIHR Nottingham Biomedical Research Centre (BRC), Nottingham, UK

**Keywords:** US, MRI, inter-rater, intra-rater, reliability

## Abstract

**Objectives:**

To systematically review the literature on inter- and intra-rater reliability of scoring US and MRI changes in hand OA.

**Methods:**

MEDLINE, EMBASE, CINHAL, Web of Science and AMED were searched from inception to January 2020. Kappa (κ), weighted kappa (κ_w_) and intra-class correlation coefficients for dichotomous, semi-quantitative and summated scores, respectively, and their 95% CI were pooled using a random-effects model. Heterogeneity between studies was assessed and reliability estimates were interpreted using the Landis–Koch classification.

**Results:**

Fifty studies met the inclusion criteria (29 US, 17 MRI, 4 involving both modalities). The pooled κ (95% CI) for inter-rater reliability was substantial for US-detected osteophytes [0.66 (0.54, 0.79)], grey-scale synovitis [0.64 (0.32, 0.97)] and power Doppler [0.76, (0.47, 1.05)], whereas intra-rater reliability was almost perfect for osteophytes [0.82 (0.80, 0.84)], central bone erosions (CBEs) [0.83 (0.78, 0.89)] and effusion [0.83 (0.74, 0.91)], and substantial for grey-scale synovitis [0.64 (0.49, 0.79)] and power Doppler [0.70 (0.59, 0.80)]. Inter-rater reliability for dichotomous assessment was substantial for MRI-detected CBEs [0.75 (0.67, 0.83)] and synovitis [0.69 (0.51, 0.87)], slight for osteophytes [0.14 (0.04, 0.25)], and almost perfect for sum score of osteophytes, CBEs, joint space narrowing (JSN), and bone marrow lesions (BMLs) (0.81–0.89). Intra-rater reliability was almost perfect for sum score of MRI synovitis [0.92 (0.87, 0.96)], BMLs [0.88 (0.78, 0.98)], osteophytes [0.86 (0.74, 0.98)], CBEs [0.83 (0.66, 1.00)] and JSN [0.91 (0.87, 0.91)].

**Conclusion:**

US and MRI are reliable in detecting hand OA features. US may be preferred due to low cost and increasing availability.

**Rheumatology key messages**

- Both ultrasound and MRI have comparable reliability in detecting imaging features of hand OA.
- Reliability for semi-quantitative score is comparable to dichotomous score and should be used preferentially as it provides greater granularity.
- Rheumatology and imaging trained assessors have comparable reliability for scoring ultrasound and MRI features of hand OA.

## Introduction

Symptomatic hand OA is common among community dwelling adults, and its prevalence increases with increasing age [1, 2]. People with hand OA often experience pain, stiffness and impaired function [3–5]. Just as for other forms of arthritis, imaging is central to understanding the disease course, outcome and pathophysiology of hand OA. EULAR recommends imaging in hand OA if there is an unexpected rapid progression of symptoms or change in clinical characteristics, with plain radiographs as the first line of imaging investigation [6]. However, plain radiographs are limited by inability to visualize synovial changes that are apparent on other imaging modalities such as US and MRI, as well as osseous changes, e.g. bone marrow lesions (BMLs), that are assessable on MRI [7].

In the past two decades, US and MRI have been used extensively to assess hand OA. US provides an inexpensive, safe and non-invasive means of assessing changes such as joint effusion, grey-scale synovitis (GSS), hyper-vascularity, osteophytes and erosions [8, 9], while MRI has the additional advantage of demonstrating BMLs [10]. However, MRI is relatively expensive and most MRI coils have limited field of view and can only image the second to fifith distal and proximal interphalangeal joints (DIPJs and PIPJs) of one hand at a time.

Though several methods to score changes in hand OA have been developed for both US [11] and MRI [12, 13] varying levels of reliability have been summarized for US in a previous narrative systematic review [14]. The reliability of assessment of features of hand OA using MRI has not been systematically reviewed and the reliability of these two imaging modalities in detecting hand OA changes has not been compared. Therefore, we aimed to systematically review the intra- and inter-rater reliability of US and MRI in detecting changes of hand OA.

## Methods

### Literature search

One reviewer (A.D.O.) performed a systematic literature search in MEDLINE, EMBASE, CINAHL, AMED and Web of Science databases from their inception until April 2018 and updated it on 14 January 2020. The search strategy was designed to capture observational studies utilizing US or MRI to examine hand OA changes. Keywords were ‘US’, ‘MRI’, ‘hand’, ‘OA’, ‘synovial effusion’, ‘synovial hypertrophy’, ‘grey-scale synovitis’, ‘synovitis’, ‘power Doppler’ (PD), ‘bone marrow lesions’ (BMLs), ‘osteophytes’, ‘joint space narrowing’ (JSN), and ‘central bone erosions’ (CBEs), their synonyms and closely related words. Details of the search strategy are provided in Supplementary Table S1, available at *Rheumatology* online. The search protocol was registered in PROSPERO (CRD42018095677).

### Inclusion and exclusion criteria

Studies were selected if they utilized US or MRI to investigate hand OA and reported inter- or intra-rater reliability. Studies investigating people with other forms of arthritis, e.g. rheumatoid or psoriatic arthritis, and non-human studies were excluded. Conference abstracts were excluded since they contain insufficient data for the purposes of a systematic review. No language restrictions were applied in the search.

### Data extraction and outcome measures

Information extracted included publication year, country, diagnostic criteria, study design, method of selecting joints or participants for reliability assessment, imaging method(s), joints assessed, scoring method(s), training background of assessor(s), and reliability measures such as kappa coefficient [weighted (κ_w_) and unweighted (κ)], intra-class correlation coefficient (ICC) and 95% CI. US and MRI features examined included osteophytes, CBE, JSN, effusion and synovitis. PD changes and MRI-detected BMLs were also examined. Where multiple publications reported reliability estimates using the same assessor(s), either the earliest publication or the publication with the most comprehensive data was included in this review. For publications providing reliability results for multiple assessors separately, each assessor’s reliability result was considered as separate data points. Where a publication used multiple assessors and reported average reliability coefficients for two sessions, data from only the first session was included if they used the same set of assessors for both sessions, or both sessions were included as separate data points if they did not use exactly the same set of assessors. For studies reporting reliability coefficient without 95% CIs, we utilized a meta-analysis effect size calculator to estimate 95% CIs given sample size and correlation coefficient [15].

### Quality assessment

The Newcastle–Ottawa scale (NOS) for case–control and cohort studies [16] and a modified NOS for cross-sectional studies [17] were used for quality assessment. This uses the star system, ranging from 0–9 stars for case–control and cohort studies, and 0–10 stars for cross-sectional studies. A high number of stars denotes good quality.

### Validation methods

The screening of titles, abstracts and full-texts, data extraction and risk of bias assessment were performed by one reviewer (A.D.O.). Two second reviewers (J.K. and S.S.), already trained in systematic review methods, independently repeated the assessments on a randomly selected sample for validation. J.K. screened titles and abstracts of 100 citations. S.S. screened full texts, assessed risk of bias and extracted data on 10% (*n* = 18), 20% (*n* = 6) and 10% (*n* = 5) of eligible articles, respectively. Discrepancies were discussed and resolved with the senior author (A.A.).

### Statistical analysis

Data analysis was performed using Stata/SE v16.1 (StataCorp, College Station, TX, USA). Reliability estimates and 95% CIs were pooled using the random-effects model with restricted maximum-likelihood method. Publication bias was assessed using Egger’s test [18]. Heterogeneity was assessed using the *I*^2^ test (0–40%: not important; 30–60%: moderate; 50–90%: substantial; 75–100%: considerable) [19]. Subgroup analyses were also performed based on scoring methods, assessors’ training background, equipment and assessment type (i.e. US assessment on dynamic scan *vs* stored images). Assessors’ training background was categorized into rheumatology (including rheumatologists and rheumatology fellows or trainees) and non-rheumatology (radiologists, radiographers, etc.). If one assessor performed assessment under the supervision of an experienced assessor, the training background of that experienced assessor was used as the training background for that assessment. Interpretation of reliability coefficients followed the Landis–Koch classification (0: poor; 0.01–0.2: slight; 0.21–0.4: fair; 0.41–0.6: moderate; 0.61–0.8: substantial; 0.81–1.0: almost perfect) [20], and systematic review outcomes were reported following PRISMA guidelines [21].

## Results

### Study selection

**Fig. 1:**
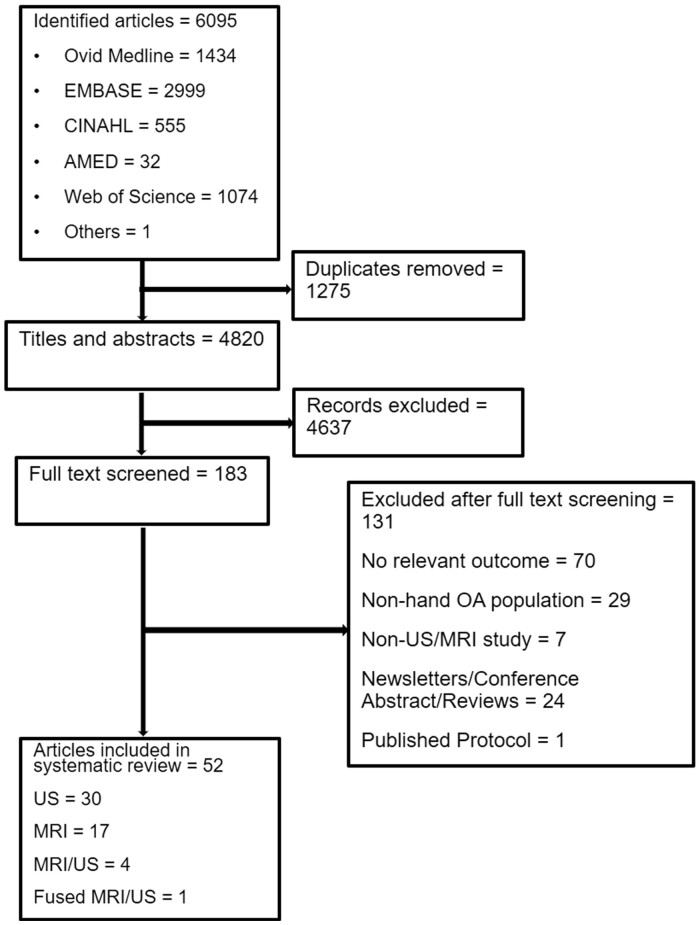
Literature search and screening flow diagram

Our search identified 6095 citations, of which 183 citations were selected for full-text review after screening of titles and abstracts. Fifty-two studies met the inclusion criteria. However, two studies were later excluded because one performed reliability assessment on fusion of US and MRI images (fusion imaging) [22] and the other performed reliability on finger, knee, hip and ankle joints, and did not provide separate estimates for individual joint [23]. This left 50 studies for inclusion in the final analysis (29 used US only, 17 used MRI only and four involved both imaging modalities.) The literature search and screening flowchart is presented in Fig. 1. Agreements between A.D.O. and the second reviewers for screening procedures and risk of bias assessment were all excellent.

### Study characteristics

Fifty articles published between 2005 and 2020 were included in this review, consisting of 240 (3654 joints) and 130 (932 joints) participants for US and MRI assessments, respectively. The majority of studies (*n* = 44) recruited participants from specialist hospital clinics and used the ACR and/or plain radiographic criteria (*n* = 38). Participants or images used for reliability assessment were selected randomly in 15 studies, serially in 11 studies, and selected to represent disease severity in four studies, while this was unclear in 20 studies. The majority of studies utilized the outcome measures in rheumatology (OMERACT) definitions for US (22 of 33) and MRI (13 of 21) pathologies. The US probes used across studies had a minimum frequency of 5 MHz and a maximum frequency of 22 MHz, with images acquired using frequency range of 11–18 MHz in most of the studies (31 of 33). One study acquired images using a frequency of 22 MHz [24], while this was unclear in one study [25]. MRI scanner strengths ranged from 1.0 to 3.0 T and the majority of MRI studies (18 of 21) performed assessment of synovitis on contrast-enhanced scans (Supplementary Tables S2, available at *Rheumatology* online). The median quality scores for risk of bias were 8 (0–9 scale) for cohort, 6 (0–9 scale) for case–control and 6 (0–10 scale) for cross-sectional studies (Supplementary Table S3, available at *Rheumatology* online).

### Inter-rater reliability of assessment of US features of hand OA

The 14 US studies that reported inter-rater reliability [11, 25–37] provided data for osteophytes (*n* = 8), JSN (*n* = 2), CBE (*n* = 2), effusion (*n* = 2), GSS (*n* = 11) and PD (*n* = 7). The pooled κ (95% CI) was substantial for dichotomously assessed osteophytes [0.66 (0.54, 0.79), *n* = 4 [11, 26, 27, 35]], JSN [0.74 (0.55, 0.93), *n* = 2 [27, 30]], GSS [0.64 (0.32, 0.97), *n* = 3 [11, 33, 35]] and PD [0.76 (0.47, 1.05), *n* = 3 [11, 34, 37]]. Similar levels of reliability were found for semi-quantitative assessment [κ_w_ (95% CI)] of osteophytes [0.69 (0.49, 0.89), *n* = 4 [11, 27, 31, 36]], GSS [0.64 (0.37, 0.90), *n* = 4 [11, 29, 36, 37]] and PD signal [0.75 (0.42, 1.08), *n* = 3 [11, 34, 37]]. Heterogeneity between studies was considerable in these analyses. A significant publication bias was present for only PD (Table 1). Reliability data were not pooled for CBE and effusion due to insufficient data reporting, use of variable outcome measures and use of same assessors in multiple studies. However, inter-rater agreement ranged from substantial to almost perfect for effusion and CBEs [34, 35]. Inter-rater reliability estimates from individual studies are presented in Supplementary Table S4, available at *Rheumatology* online.

**Table 1:** Pooled estimates for inter-rater reliability of detecting US features of hand OA

Pathology	No. of studies	No. of participants	No. of joints	Point estimate (95% CI)	Heterogeneity		Pub. bias
					*I* ^2^ (%)	*P*-value	*P*-value
Kappa (unweighted) for dichotomous							
Osteophytes	4	36	745	0.66 (0.54, 0.79)	85.13	<0.001	0.83
Joint space abnormality	2	13	165	0.74 (0.55, 0.93)	71.63	0.06	—
Grey scale synovitis	3	27	585	0.64 (0.32, 0.97)	92.43	<0.001	0.36
Power Doppler	3	21	215	0.76 (0.47, 1.05)	85.81	0.01	0.004
Kappa (weighted) for categorical data							
Osteophytes	4	32	425	0.69 (0.49, 0.89)	98.92	<0.001	0.07
Grey scale synovitis	4	32	375	0.64 (0.37, 0.90)	93.11	<0.001	0.59
Power Doppler	3	16	265	0.75 (0.42, 1.08)	97.87	0.03	0.009

Pub.: publication.

### Intra-rater reliability of assessment of US features of hand OA

Intra-rater reliability of assessment of US features of hand OA was reported in 26 studies [7, 11, 24, 25, 27, 30–33, 38–54]. GSS was the most investigated feature (*n* = 21), followed by PD (*n* = 18), osteophytes (*n* = 14), effusion (*n* = 8), JSN (*n* = 5) and CBE (*n* = 3). The pooled reliability coefficient was almost perfect for dichotomous assessment [κ (95% CI)] of osteophytes [0.82 (0.80, 0.84), *n* = 5 [11, 24, 39, 40, 52]], CBEs [0.83 (0.78, 0.89, *n* = 2 [49, 52]] and effusion [0.83 (0.74, 0.91), *n* = 4 [44, 49, 50, 52]], and substantial for GSS [0.64 (0.49, 0.79), *n* = 6 [11, 33, 44, 49, 50, 52]] and PD [0.70 (0.59, 0.80, *n* = 6 [11, 44, 49, 50, 52, 54]]. Intra-rater agreement for semi-quantitative assessment [pooled κ_w_ (95% CI)] was almost perfect for osteophytes [0.87 (0.81, 0.94), *n* = 4 [11, 31, 53, 54]] and substantial for GSS [0.73 (0.64, 0.82), *n* = 5 [11, 32, 40, 53, 54]] and PD [0.67 (0.53, 0.82), *n* = 4 [11, 32, 40, 53]], while assessment of sum score [pooled ICC (95% CI)] was almost perfect for GSS [0.83 (0.71, 0.96), *n* = 3 [33, 38, 43]] and substantial for PD [0.61 (0.53, 0.68), *n* = 2 [38, 43]]. Heterogeneity between studies was considerable in these analyses, except for dichotomous assessment of osteophytes and sum score of PD, which were unimportant (Table 2). Analysis for intra-rater reliability for assessment of JSN was not performed due to insufficient data and heterogeneity in the report pattern. For example, of five studies that reported this, one reported 100% agreement for DIPJs and PIPJs [51], two reported substantial agreement (κ = 0.64) using the same cohort and assessors [39, 40], and two reported κ range for cartilage abnormalities [27, 30]. Details are available in Supplementary Table S5, available at *Rheumatology* online.

**Table 2:** Pooled estimates for intra-rater reliability of detecting US features of hand OA

Pathology	No. of studies	No. of participants	No. of joints	Point estimate (95% CI)	Heterogeneity		Pub. bias
					*I* ^2^ (%)	*P*-value	*P*-value
Kappa (unweighted) for dichotomous							
Osteophytes	5	33	975	0.82 (0.80, 0.84)	0.02	0.04	0.02
Erosion	2	37	930	0.83 (0.74, 0.91)	69.45	0.07	—
Effusion	4	78	2090	0.83 (0.78, 0.89)	90.59	<0.001	0.85
Grey scale synovitis	6	94	1940	0.64 (0.49, 0.79)	93.72	<0.001	<0.00001
Power Doppler	6	119	2154	0.70 (0.59, 0.80)	81.48	<0.001	0.007
Kappa (weighted) for categorical data							
Osteophytes	4	71	495	0.87 (0.81, 0.94)	97.70	<0.001	0.02
Grey scale synovitis	5	71	748	0.73 (0.64, 0.82)	80.66	<0.001	0.15
Power Doppler	4	51	635	0.67 (0.53, 0.82)	80.98	<0.001	0.0001
Intra-class correlation coefficient for continuous data							
Grey scale synovitis	3	26	285	0.83 (0.71, 0.96)	85.23	<0.001	0.35
Power Doppler	2	11	270	0.61 (0.53, 0.68)	0.01	0.57	—

Pub.: publication.

### Inter-rater reliability of assessment of MRI features of hand OA

Inter-rater reliability of assessment of MRI features of hand OA was examined in 12 studies [12, 13, 35, 55–63]. CBE was the most investigated feature (*n* = 12), followed by osteophytes (*n* = 10), synovitis (*n* = 9), JSN (*n* = 8), BMLs (*n* = 7) and then effusion (*n* = 1). The pooled reliability coefficient was substantial for dichotomous assessment [κ (95% CI] of CBEs [0.75 (0.67, 0.83), *n* = 3 [35, 56, 58]] and synovitis [0.69 (0.51, 0.87), *n* = 2 [35, 58]] but poor for osteophytes [0.14 (0.04, 0.25), *n* = 2 [35, 58]]. Conversely, the pooled reliability estimate for sum score [ICC (95% CI)] was almost perfect for osteophytes [0.81 (0.65, 0.96), *n* = 5 [12, 13, 57, 59, 60]], CBEs [0.89 (0.79, 0.99), *n* = 4 [12, 13, 57, 60]], JSN [0.86 (0.69, 1.03), *n* = 4 [12, 13, 57, 60]] and BMLs [0.84 (0.71, 0.96), *n* = 6 [12, 13, 57, 59–61]], and substantial for synovitis [0.65 (0.52, 0.79), *n* = 6 [12, 13, 57, 59–61]]. Heterogeneity was considerable for sum score of osteophytes, JSN and BMLs but moderate for erosion and synovitis. Only studies involved in meta-analyses of erosion and BMLs had significant publication bias (Table 3). One study examined effusion and reported a moderate agreement for dichotomous assessment (κ = 0.41–0.54) [35]. Data from individual studies are presented in Supplementary Table S6, available at *Rheumatology* online.

**Table 3:** Pooled estimates for inter-rater reliability of detecting MRI features of hand OA

Pathology	No. of studies	No. of participants	No. of joints	Point estimate (95% CI)	Heterogeneity		Pub. bias
					*I* ^2^ (%)	*P*-value	*P*-value
Kappa (unweighted) for dichotomous							
Osteophytes	2	23	184	0.14 (0.04, 0.25)	0.00	0.82	—
Erosion	3	38	199	0.75 (0.67, 0.83)	0.00	0.45	0.29
Synovitis	2	23	184	0.69 (0.51, 0.87)	70.13	0.07	—
Intra-class correlation coefficient for continuous data							
Osteophytes	5	69	412	0.81 (0.65, 0.96)	86.12	<0.001	0.63
Erosion	4	49	392	0.89 (0.79, 0.99)	50.47	0.04	0.005
Joint space narrowing	4	49	392	0.86 (0.69, 1.03)	89.31	<0.001	0.18
Bone marrow lesions	6	134	502	0.84 (0.71, 0.96)	95.00	<0.001	<0.00001
Synovitis	6	118	486	0.65 (0.52, 0.79)	49.89	0.05	0.15

Pub.: publication.

### Intra-rater reliability of assessment of MRI features of hand OA

**Fig. 2:**
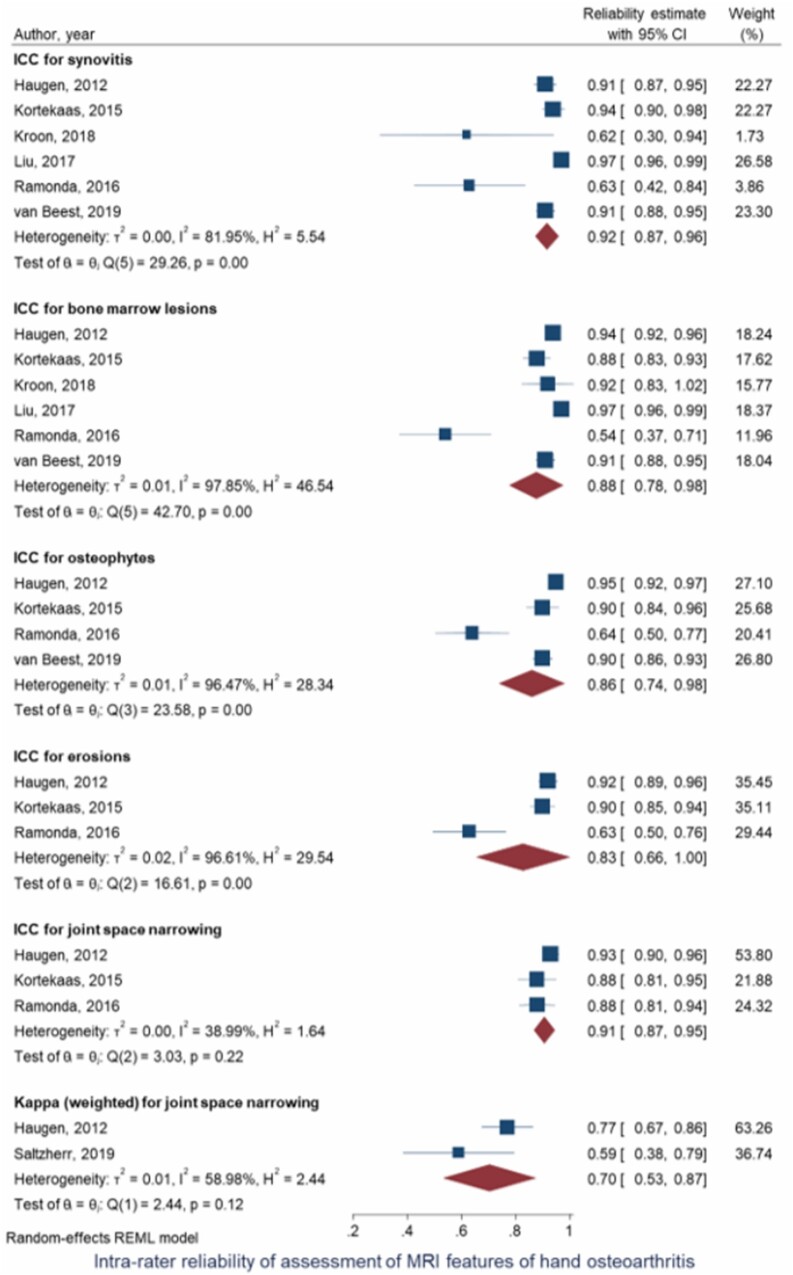
Forest plot of intra-rater reliability of detecting MRI features of hand OA ICC: intra-class correlation coefficient.

Fourteen studies examined Intra-rater reliability of assessment of MRI features of hand OA [7, 10, 29, 48, 55, 59, 60, 62–68]. BMLs and synovitis were the most examined features (*n* = 10), followed by osteophytes and CBEs (*n* = 8), JSN (*n* = 7) and effusion (*n* = 1). The pooled reliability estimate for sum score [ICC (95% CI)] was almost perfect for synovitis [0.92 (0.87, 0.96), *n* = 6 [7, 10, 48, 60, 65, 68]], BMLs [0.88 (0.78, 0.98), *n* = 6 [7, 10, 48, 60, 65, 68]], osteophytes [0.86 (0.74, 0.98), *n* = 4 [7, 60, 65, 68]], CBEs [0.83 (0.66, 1.00), *n* = 3 [7, 60, 65]] and JSN [0.91 (0.87, 0.95), *n* = 3 [7, 60, 65]], whereas agreement for semi-quantitative assessment of JSN was substantial [κ_w_ (95% CI): 0.70 (0.53, 0.87), *n* = 2 [62, 65]]. Heterogeneity between studies was considerable and there was significant publication bias for all MRI features in these analyses except JSN (Fig. 2). Data for intra-rater reliability from individual studies are presented in Supplementary Table S7, available at *Rheumatology* online.

### Subgroup analyses based on assessors’ training background, assessment type, and equipment

Inter-rater agreement for binary score of osteophytes on US was comparable for raters with a training background in rheumatology [κ (95% CI): 0.70 (0.46, 0.95), *n* = 2 [27, 35]] than raters with non-rheumatology training background [κ (95% CI): 0.61 (0.51, 0.71), *n* = 2 [11, 26]]. However, heterogeneity was considerably high for the rheumatology trained assessors (*I*^2^ = 95.83%) and unimportant for non-rheumatology trained assessors (*I*^2^ = 0.00%). Similar results were observed for intra-rater agreement of semi-quantitative assessment of GSS [κ_w_ (95% CI): 0.75 (0.65, 0.86) with *I*^2^ = 79.59%, *n* = 4 [11, 32, 40, 54] and 0.66 (0.59, 0.72) with *I*^2^ = 0.00%, *n* = 2 [11, 53], respectively]. No significant difference was observed for binary score of osteophytes between the two groups. Conversely, intra-rater agreement for assessment of PD signal was numerically better for non-rheumatology trained assessors [κ (95% CI): 0.78 (0.64, 0.92) with *I*^2^ = 0.01%, *n* = 2 [11, 52]] than rheumatology trained assessors [κ (95% CI): 0.67 (0.53, 0.80) with *I*^2^ = 76.25%, *n* = 5 [11, 44, 49, 50, 54]]. Furthermore, intra-rater agreement was slightly better for assessment of osteophytes, GSS and PD signal on stored images than on real-time scan, and better for assessment of osteophytes and GSS on images acquired with scan frequencies ≥15 MHz than with scan frequencies ≤14 MHz (Supplementary Table S8, available at *Rheumatology* online).

Inter-rater reliability for assessment of MRI synovitis and BMLs was numerically better for raters with a training background in rheumatology [ICC (95% CI): 0.79 (0.62, 0.96) and 0.89 (0.83, 0.96), *n* = 2 [12, 61], respectively] than raters with non-rheumatology training background [ICC (95% CI): 0.59 (0.42, 0.77) and 0.82 (0.64, 1.00), *n* = 5 [13, 57, 59–61]]. Similar findings were observed for intra-rater reliability for assessment of MRI synovitis and BMLs [ICC (95% CI): 0.92 (0.90, 0.94) and 0.92 (0.89, 0.95), *n* = 4 [7, 48, 65, 68] for rheumatology trained assessors and 0.82 (0.48, 1.15) and 0.76 (0.34, 1.18), *n* = 2 [10, 60] for non-rheumatology trained assessors]. In these analyses, heterogeneity was unimportant to moderate for rheumatology trained assessors and moderate to considerable for non-rheumatology trained assessors. The OMERACT scoring method produced a higher level of reliability for BML and synovitis with non-significant heterogeneity than the Oslo scoring method. Additionally, reliability assessment of synovitis, osteophytes and CBE was better for assessments performed on images acquired with 1.0 T than 1.5 T scanners (Table 4).

**Table 4:** Subgroup analysis for reliability of detecting MRI features of hand OA

Pathology	Assessors' training background		Scoring method		Scanner strength	
	Rheumatology	Non-rheumatology	OMERACT	Oslo	1.0 T	1.5 T
Inter-rater reliability: ICC (95% CI) [*I*^2^], number of studies						
Synovitis	0.79 (0.62, 0.96) [0.00%], *n* = 2	0.59 (0.42, 0.77) [52.39%], *n* = 5	0.73 (0.60, 0.86) [9.75%], *n* = 4	0.60 (0.36, 0.85) [64.24%], *n* = 4	0.74 (0.61, 0.87) [15.13%], *n* = 4	0.56 (0.33, 0.79) [48.55%], *n* = 2
Bone marrow lesion	0.89 (0.83, 0.96) [0.01%], *n* = 2	0.82 (0.64, 1.00) [96.54%], *n* = 5	0.93 (0.86, 0.99) [74.50%], *n* = 4	0.73 (0.47, 1.00) [87.89%], *n* = 4		
Osteophyte	—	0.79 (0.60, 0.97) [88.73%], *n* = 5	0.81 (0.65, 0.98) [0.00%], *n* = 2	0.81 (0.59, 1.03) [92.63%], *n* = 4	0.94 (0.87, 1.01) [19.22%], *n* = 3	0.65 (0.31, 0.99) [88.56%], *n* = 2
Joint space narrowing	—	0.80 (0.60, 1.01) [82.00%], *n* = 3	—	0.83 (0.60, 1.06) [91.47%], *n* = 3	—	—
Erosion	—	0.80 (0.59, 1.01) [74.91%], *n* = 4	—	0.88 (0.75, 1.01) [67.72%], *n* = 4	0.91 (0.80, 1.01) [0.01%], *n* = 2	0.56 (0.14, 0.97) [67.47], *n* = 2
Intra-rater reliability: ICC (95% CI) [*I*^2^], number of studies						
Synovitis	0.92 (0.90, 0.94) [0.05%], *n* = 4	0.82 (0.48, 1.15) [90.49%], *n* = 2	0.81 (0.54, 1.08) [67.92%], *n* = 2	0.85 (0.69, 1.01) [95.59%], *n* = 3		—
Bone marrow lesion	0.92 (0.89, 0.95) [46.52%], *n* = 4	0.76 (0.34, 1.18) [95.90%, *n* = 2]	0.91 (0.88, 0.94) [0.01%], *n* = 2	0.80 (0.57, 1.03) [98.12%], *n* = 3		—
Osteophytes	0.92 (0.88, 0.96) [65.41%], *n* = 3	—	—	0.84 (0.74, 1.02) [96.18%]	—	—
Erosion	0.91 (0.88, 0.94) [0.00%], *n* = 2	—	—	0.83 (0.66, 1.00) [96.61%], *n* = 3	—	—

*I* ^2^: heterogeneity; ICC: intra-class correlation coefficient.

### Sensitivity analysis

All US studies either assessed reliability on the same stored image or on real-time scans repeated on the same day, with the notable exception of two studies that assessed on real-time scans repeated after 1 week [49] and 12 weeks [50]. Data from for these two studies were excluded in a sensitivity analysis for inflammatory features. There was no significant difference with the two studies excluded, except for GSS where reliability reduced from substantial to moderate (Supplementary Table S9, available at *Rheumatology* online). Furthermore, contrast enhancement was used in all MRI studies involved in the analysis for synovitis, except one study [48]. There was no observable difference in the results when this study was excluded from the analysis (Supplementary Table S10, available at *Rheumatology* online).

## Discussion

This is the first comprehensive systematic review and meta-analysis of the reliability of US and MRI in detecting features of hand OA. The key findings of this study are: (i) agreement was moderate to almost perfect for US; and (ii) agreement was slight to almost perfect for MRI features of hand OA. Our findings for inter- and intra-rater reliability of assessment of US features were consistent with those reported in a previous meta-analysis for knee OA [14].

Generally, intra-rater reliability was higher than inter-rater reliability for both US and MRI. Reliability was numerically lower for US than MRI, particularly for synovitis. However, inter-rater agreement for binary scoring of osteophytes was slight for MRI but substantial for US. This finding should be interpreted with care since only two studies were involved in the pooled estimate for MRI-detected osteophytes. US is widely perceived as an operator-dependent technique, and several factors such as probe positioning, acquisition of images in real-time, and interpretation of the acquired images affect reliability [69, 70]. This may explain why reliability for PD signal, GSS and osteophyte was better for US imaging studies that used static images than those that used real-time scan (Supplementary Table S8, available at *Rheumatology* online).

Reliability was comparable when dichotomous and semi-quantitative scores were used. However, reliability was best for summated scores, particularly for MRI assessed JSN, osteophytes and CBE. Reliability assessment of imaging features using summated score could potentially be overoptimistic, since it is based on the sum of grades of a pathology in the whole hand without accounting for the individual joints that are affected. For example, if rater A scores as grade 2 a pathology in the second to fifth PIPJs in one participant and rater B scores as grade 2 a pathology in the second to fifth DIPJs of the same participant, agreement between the two raters will be almost perfect for summated score but poor for dichotomous and semi-quantitative assessments.

The frequency range used for B-mode scan across studies ranged from 11 to 18 MHz, except for one study that used a high resolution probe with frequency up to 22 MHz [24]. Reliability estimates for osteophytes and synovitis were better for assessment on images acquired with scan frequency ≥15 MHz than frequency ≤14 MHz. It is noteworthy that as scan frequency increases, spatial resolution increases but tissue penetration reduces [71, 72], which makes high-frequency probes suitable for scanning joints that are superficial such as finger joints. Conversely, MRI scanner strength used across studies ranged from 1.0 to 3.0 T. Inter-rater reliability of detection of MRI synovitis, osteophytes and CBE was better for assessments performed on images acquired with 1.0 T than 1.5 T scanners. Of three studies that utilized 3.0 T scanners to examine osteophytes, inter-rater reliability was slight in two [35, 58], but almost perfect in one study [59]. Both studies with slight agreement performed dichotomous assessment whereas the latter performed sum score assessment. It is noteworthy that the experience of the raters could also contribute to the variable reliability. Across the three studies, only one stated years of experience of the raters, which was 12–13 years [58]. Therefore, further studies are required to explore the impact of scanner quality on the reliability of detecting features of hand OA.

Furthermore, subgroup analyses based on training background showed that reliability assessments of US and MRI features of hand OA were broadly comparable for rheumatology and imaging trained assessors. There were subtle numeric differences for some imaging features, but these were not significantly different. These findings suggest that rheumatology trained assessors may be sufficient to undertake research and clinical assessments using US or MRI in people with hand OA.

Several studies have adopted the OMERACT definitions [73] and semi-quantitative scoring methods for US pathologies in hand joints [74], which were originally developed for RA but adapted for assessment of hand OA. This has been criticized as it could contribute to a floor effect when assessing inflammatory changes [14] since inflammation is only present at a low level in hand OA [44], necessitating development of scoring systems tailored to hand OA. Keen and colleagues have developed a preliminary scoring system for US features of hand OA [11], which is gaining widespread usage. However, this is not accompanied with representative images for reference purposes, while a few atlases have been developed for scoring of osteophytes [31] and cartilage damage [27].

There are potential limitations to this review. Firstly, we focused only on hand OA. Therefore, findings are not generalizable to OA in other joints. However, our findings are consistent with those of previous meta-analysis on knee OA [14]. Secondly, there was significant heterogeneity across studies included in the analyses. Therefore, caution should be applied when interpreting findings from this review. Nevertheless, this review highlights the reliability of US and MRI in detecting features of hand OA. Additionally, it highlights a lack of representative imaging atlas devised for most US features of hand OA.

In conclusion, both US and MRI are reliable in detecting hand OA changes. However, further standardization of techniques and development of representative atlases for all imaging features of hand OA are essential.

## Supplementary Material

keab470_supplementary_data
