# Supplementary material for: Reliability of detection of ultrasound and MRI features of hand osteoarthritis: a systematic review and meta-analysis
Source: Rheumatology (Oxford). 2021 Jun 4;61(2):542–53. doi: 10.1093/rheumatology/keab470 (PMC8824416; doi:10.1093/rheumatology/keab470)
Supplement: keab470_supplementary_data [file keab470_supplementary_data.docx]

**Supplementary Table S1: Search strings**

| Database | Search strategy |
| --- | --- |
|  |  |
| Ovid MEDLINE | 1 (Hand* or Hand joint* or finger* or finger joint* or thumb or thumb base or wrist or wrist joint*).mp. [mp=title, abstract, original title, name of substance word, subject heading word, keyword heading word, protocol supplementary concept word, rare disease supplementary concept word, unique identifier, synonyms]  2 (Interphalan* interphalangeal joint* or metacarpophalang* metacarpophalangeal joint* or metacarpus or carpometacarp* or carpometacarpal joint).mp.  3 (Trapeziometacarp* or TMC joint or scaphotrapez* or ST joint or DIP or PIP or MCP or CMC).mp.  4 1 or 2 or 3  5 Osteoarthritis.mp. or exp OSTEOARTHRITIS/  6 (Degenerative arthritis or generalised osteoarthritis or erosive osteoarthritis or non-erosive osteoarthritis).mp. (1313)  7 (OA or osteophyte* or pain or JSN or heberden or bouchard or effusion or hypertrophy or cartilage thickness or symptom*).mp.  8 Bone Marrow Lesion.mp.  9 Joint Space Narrowing.mp.  10 5 or 6 or 7 or 8 or 9  11 4 and 10  12 (Hand osteoarthritis or Hand OA or HOA).mp.  13 11 or 12  14 (Ultrasonograph* or Sonograph* or Ultrasound).mp  15 (Doppler or Dopplerography or gr?y scale or gr?y-scale synovitis or PDS or PD or GS synovitis).mp.  16 Gray scale synovitis.mp.  17 Power Doppler Signal.mp.  18 14 or 15 or 16 or 17  19 Magnetic Resonance Imaging/  20 (MRI or CE MRI or fat saturation or spin echo or SE or gradient echo or GE or PDW).mp.  21 (Contrast enhanced or non-contrast enhanced or dynamic contrast-enhanced).mp.  22 Proton Density weighted.mp.  23 19 or 20 or 21 or 22  24 18 or 23  25 13 and 24  26 Cohort Studies/  27 Cohort$.tw.  28 Epidemiologic Methods/  29 (Case$ and Control$).tw.  30 Cross-Sectional Studies/  31 Observational Study/  32 Case-Control Studies/  33 Prevalence studies.mp.  34 Relative risk.mp.  35 Odds Ratio/  36 Hazard ratio.mp.  37 26 or 27 or 28 or 29 or 30 or 31 or 32 or 33 or 34 or 35 or 36  38 25 and 37  39 limit 38 to (humans and "all adult (19 plus years)") |
|  |  |

**Supplementary Table S2a: Ultrasound study characteristics**

| **Author, year** | **Country** | **Design** | **Setting** | **Diagnosis** | **Selection** | **Size (N)** | **Joints (N)** | **Joints list** | **Scan type** | **Probe frequency (MHz)** | **Assessors** |
| --- | --- | --- | --- | --- | --- | --- | --- | --- | --- | --- | --- |
| Abraham, 2014 | UK | Cross-sectional | Community | People born in 1947 | Unclear | 25 | 100 | CMC, MCP, PIP, DIP | Static | 1. o 18 | 1-Rheumatology Teaching fellow (MSK US trained)  2-Unclear (MSK US trained) |
| Damman, 2016 | Netherlands | Cohort | Rheumatology OPC | ACR | Random | 6 | 180 | CMC, MCP, PIP, DIP | Dynamic | 10 to 14 | 1-Rheumatologist (supervised scan)  2-PhD student (under Kloppenburg) |
| Dejaco, 2017 | Austria | Cross-sectional | Clinical routine practice | ACR | Consecutive | 9 | 324 | MCP, PIP, DIP plus non-hand joints | Static | 6 to 18 | Rheumatologist |
| Hammer, 2016 | Norway | Cross-sectional | Rheumatology OPC | ACR | Unclear | 5 | 40 | MCP | Dynamic | 6 to 18 | 9 Rheumatologists + 1 Rheumatology research fellow |
| Keen, 2008a | UK | Cross-sectional | MSK clinic | ACR or symptomatic + radiographic | Consecutive | 10 | 240 | CMC, MCP, PIP, DIP | Dynamic | 7 to 15 | Rheumatology research fellow |
| Iagnocco, 2005 | Italy | Cross-sectional | Rheumatology OPC | ACR + radiographic OA | Unclear | Unclear | Unclear | DIP, PIP | Unclear | 8 to 16 | 2 Rheumatologists |
| Keen, 2008b | France | Cross-sectional | Rheumatology OPC | Unclear | Unclear | 7 | 105 | CMC, MCP, PIP, DIP | Dynamic | 8 to 14 | 5 Rheumatologists and 2 Radiologists |
| Haugen, 2016 | Norway | Cohort | Rheumatology OPC | ACR | Unclear | 5 | 15 | CMC, MCP, PIP, DIP | Unclear | 5 to 13 | Rheumatology research fellow  Rheumatologist |
| Keen, 2008c | UK | Case-control | MSK clinic | ACR or symptomatic + radiographic | Unclear | 10 | 300 | CMC, MCP, PIP, DIP | Dynamic | 7 to 15 | Rheumatology research fellow |
| Iagnocco, 2012 | Italy | Cross-sectional | Unclear | ACR | Consecutive | 8 | 40 | MCP | Dynamic | 18 | 9 Rheumatologists |
| Keen, 2010 | UK | Cross-sectional | MSK clinic | ACR or symptomatic + radiographic | Unclear | 10 | 300 | CMC, MCP, PIP, DIP | Dynamic | 7 to 15 | Rheumatologist |
| Kortekaas, 2014 | Netherlands | Cross-sectional | Rheumatology OPC | ACR | Random | 6 | 180 | CMC, MCP, PIP, DIP | Dynamic | 10 to 14 | 1-Rheumatologist (experienced MSK US)  2-PhD student (experienced MSK US) |
| Kortekaas, 2013 | Netherlands | Case-control | Rheumatology OPC | ACR | Random | 5 | 90 | DIP, PIP | Dynamic | 10 to 14 | 1-Rheumatologist (supervised scan)  2-PhD student (under Kloppenburg) |
| Kortekaas, 2015a | Netherlands | Cohort | Rheumatology OPC | ACR | Random | 6 | 180 | CMC, MCP, PIP, DIP | Dynamic | 10 to 14 | 1- PhD student (experienced MSK US)  2-Rheumatologist (experienced MSK US) |
| Kortekaas, 2011 | Netherlands | Cross-sectional | Rheumatology OPC | ACR | Random | 6 | 180 | CMC, MCP, PIP, DIP | Dynamic | 10 to 14 | 1-Rheumatologist (supervised scan)  2-PhD student (under Kloppenburg) |
| Kortekaas, 2010 | Netherlands | Cross-sectional | Rheumatology OPC | ACR | Random | 6 | 180 | CMC, MCP, PIP, DIP | Dynamic | 10 to 14 | 1-Rheumatologist (supervised scan)  2-PhD student (under Kloppenburg) |
| Kortekaas, 2015b | Netherlands | Cross-sectional | Rheumatology OPC | ACR + radiographic erosion | Unclear | 6 | 48 | DIP, PIP | Unclear | 10 to 14 | 1-Rheumatologist (supervised scan)  2-PhD student (under Kloppenburg) |
| Kortekaas, 2016 | Netherlands | Cohort | Rheumatology OPC | ACR | Random | 5 | 90 | DIP, PIP | Dynamic | 10 to 14 | 1-Rheumatologist (supervised scan)  2-PhD student (under Kloppenburg) |
| Kroon, 2018 | Netherlands | Cross-sectional | Rheumatology OPC | ACR | Unclear | Un | Un | Thumb | Dynamic | 10 to 14 | 2 Ultrasonographers |
| Magnusson, 2017 | Norway | Cross-sectional | Community | Radiographic OA | Un | Un | Unclear | Unclear | Unclear | Trained medical student |  |
| Mancarella, 2015 | Italy | Cohort | Rheumatology OPC | ACR | Random | 15 | 270 | DIP, PIP | Dynamic | 11.4 | Experienced MSK US |
| Mathiessen, 2013 | Norway | Cross-sectional | Rheumatology OPC | ACR and/or Radiographic OA | Random | 10 | 150 | CMC, MCP, PIP, DIP | Static | 5 to 13 | Rheumatology research fellow (MSK US trainee)  Rheumatologist |
| Mathiessen, 2016 | Norway | Cohort | Rheumatology OPC | ACR and/or Radiographic OA | Unclear | Un | Un | CMC, MCP, PIP, DIP | Static | 5 to 13 | Rheumatology research fellow (MSK US trainee)  Rheumatologist |
| Mancarella, 2010 | Italy | Case-control | Rheumatology OPC | ACR | Consecutive | 35 | 980 | MCP, PIP, DIP | Static | 5 to 13 | Experienced MSK US |
| Paschoal, 2017 | Brazil | Cross-sectional | Rheumatology OPC | ACR | Consecutive | 15 | 15 | DIP, PIP | Dynamic | 10 to 18 | Rheumatologist (5 yrs experience in MSK US) |
| Sivakumaran, 2018 | UK | Cross-sectional | Rheumatology OPC | EULAR | Consecutive | Un | Un | CMC, MCP, PIP, DIP | Unclear | 8 to 22 | Rheumatologist (6 yrs experience in US) |
| Uson, 2014 | Spain | Cross-sectional | Rheumatology OPC | ACR | Consecutive | 5 | 80 | DIP, PIP | Video clip | 12 | Rheumatologist (MSK US expert) |
| Vlychou, 2009 | Greece | Cross-sectional | Rheumatology OPC | ACR + radiographic erosion | Consecutive | 22 | 660 | CMC, MCP, PIP, DIP | Dynamic | 8 to 13 | Radiologist (MSK US expert, 4 years’ experience) |
| Wittoek, 2010 | Belgium | Case-control | Unclear | ACR + radiographic erosion | Unclear | 10 | 180 | DIP, PIP | Static | 10 to 18 | Rheumatologist  Rheumatologist (trained MSK US) |
| Wittoek, 2011 | Belgium | Case-control | Hospital's OPC | ACR | Consecutive | 5, 14 | 90, 112 | DIP, PIP | Dynamic | 12 to 18 | Rheumatologist  Rheumatologist (trained MSK US) |
| Fjellstad, 2019 | Norway | Cross-sectional | Rheumatology OPC | Symptomatic or ultrasound osteophyte | Unclear | 10 | 100 | CMC, MCP, PIP, DIP | Dynamic | 6 to 15 | Trained medical student  Rheumatology research fellow (experienced MSK US) |
| Mattap, 2019 | Australia | Cross-sectional | Community | Unclear | Unclear | 20 | 300 | CMC, MCP, PIP, DIP | Dynamic | 8 to 18 | Radiographer |
| Oo, 2019 | Australia | Cross-sectional | Community | Thumb-base pain | Random | 40 | 40 | Thumb | Static | 12 | Rheumatology research fellow MSK US expert (4 yrs experience) |
| Petterson, 2020 | Norway | Cross-sectional | Rheumatology OPC | Symptomatic or ultrasound osteophyte | Unclear | 10 | 20 | DIP, PIP | Unclear | 6 to 15 | Trained medical student  Rheumatology research fellow (experienced MSK US) |

Size (N)- number of patients, Joints (N)-number of joints, UK-United Kingdom, CMC-carpometacarpal, MCP-metacarpophalangeal, PIP-proximal interphalangeal, DIP-distal interphalangeal, MSK US-musculoskeletal ultrasonography, OPC-outpatient clinic, ACR-American College of Rheumatology, Un-unclear,

**Supplementary Table S2b: MRI study characteristics**

| **Author, year** | **Country** | **Design** | **Setting** | **Diagnosis** | **Selection** | **Size (N)** | **Joints (N)** | **Joints list** | **Scanner** | **Use of contrast agent** | **Assessors** |
| --- | --- | --- | --- | --- | --- | --- | --- | --- | --- | --- | --- |
| Damman, 2017 | Netherlands | Cohort | Hospital's OPC | Clinical diagnosis by rheumatologist | Unclear | 11 | 88 | DIP, PIP | 1.5T | Yes | Unclear |
| Grainger, 2007 | UK | Cross-sectional | Hospital's OPC | ACR | Consecutive | 15 | 15 | DIP, PIP | 1.5T | Yes | 2 Radiologists (MSK experienced) |
| Haugen, 2011 | Norway | Cross-sectional | Rheumatology OPC | ACR | Selected to represent disease severity | 10 | 80 | DIP, PIP | 1.0T | Yes | Rheumatology research fellow |
| Haugen, 2012 | Norway | Cross-sectional | Rheumatology OPC | ACR | Random | 10 | 80 | DIP, PIP | 1.0T | Yes | Rheumatology research fellow |
| Haugen, 2012 | Norway | Cross-sectional | Rheumatology OPC | ACR | Random | 10 | 80 | DIP, PIP | 1.0T | Yes | Rheumatology research fellow |
| Haugen, 2015 | Norway | Cross-sectional | Rheumatology OPC | ACR | Selected to represent disease severity | 20 | 160 | DIP, PIP | 1.0T | Yes | Radiologist  Rheumatologist  Rheumatologist |
| Haugen, 2014 | Norway | Cross-sectional | Rheumatology OPC | ACR | Selected to represent disease severity | 8 | 64 | DIP, PIP | 1.0T | Yes | Rheumatology research fellow |
| Jans, 2013 | Belgium | Cross-sectional | Hospital's OPC | ACR + radiographic erosion | Unclear | 9 | 72 | DIP, PIP | 3.0T | Yes | Radiologist (9 years’ experience)  Radiologist (13 years’ experience) |
| Haugen, 2017 | Norway | Cohort | Rheumatology OPC | ACR | Consecutive | 7 | 48 | DIP, PIP | 1.0T | Yes | Rheumatology research fellow |
| Haugen, 2016a; 2016b | Norway | Cohort | “ | ACR | Consecutive | 5 | 40 | DIP, PIP | 1.0T | Yes | Rheumatology research fellow |
| Kortekaas, 2015 | Netherlands | Cross-sectional | Rheumatology OPC | ACR + radiographic erosion | Unclear | 6 | 48 | DIP, PIP | 3.0T | Yes | PhD student (under Kloppenburg) [Trained assessor] |
| Kroon, 2018 | Netherlands | Cross-sectional | Rheumatology OPC | ACR | Unclear | Un | Un | Thumb | 1.5T | No | Rheumatology trainees |
| Kroon, 2019 | Netherlands/ Israel | Cross-sectional | Rheumatology OPC |  | Unclear | 20 | 20 | Thumb | 1.5T/3.0T | Yes | Assessment 1: by 1 Rheumatologist and 2 rheumatology fellows  Assessment 2: by 1 Radiologist and 1 rheumatology fellow |
| Liu, 2017 | Netherlands | Cross-sectional | Rheumatology OPC | Clinical diagnosis by rheumatologist | Random | 11 | 88 | DIP, PIP | 1.5T | Yes | Rheumatology trainee  Radiologist (supervised assessment) |
| Ramonda, 2016 | Italy | Cross-sectional | Rheumatology OPC | ACR + radiographic erosion | Unclear | 11 | 88 | DIP, PIP | 1.5T | Yes | Radiologists |
| Wittoek, 2011 | Belgium | Case-control | Hospital's OPC | ACR | Consecutive | 5, 14 | 90, 112 | DIP, PIP | 3.0T | Yes | Radiologists |
| Kroon, 2019 | Netherlands | Longitudinal | Rheumatology OPC | Clinical diagnosis by rheumatologist | Selected to represent disease severity | 49 | 90 | Thumb | 1.5T | No | Rheumatologist  Rheumatology fellow  Rheumatology fellow  Radiologist |
| Saltzherr, 2019 | Netherlands | Cross-sectional | Rheumatology OPC | Rheumatologist diagnosed + radiographic OA |  | 10 | 234 | MCP, PIP | 3.0T | Yes | Radiologist (3 years’ experience)  Radiologist (10 years’ experience)  Rheumatology research fellow |
| Van Beest, 2019 | Netherlands | Cohort | Rheumatology OPC | Rheumatologist diagnosed | Random | 16 | 128 | DIP, PIP | 1.5T | Yes | Rheumatology trainees |
| Van Beest, 2019 | Netherlands | Cohort | Rheumatology OPC | Rheumatologist diagnosed | Random | 10 | 10 | Thumb | 1.5T | No | Rheumatology trainees |

Size (N)- number of patients, Joints (N)-number of joints, UK-United Kingdom, CMC-carpometacarpal, MCP-metacarpophalangeal, PIP-proximal interphalangeal, DIP-distal interphalangeal, MSK US-musculoskeletal ultrasonography, OPC-outpatient clinic, ACR-American College of Rheumatology, MRI-magnetic resonance imaging, NA-not applicable

**Supplementary Table S3-Quality assessment**

***Supplementary Table S3a: Quality Assessment for Cohort Studies***

| Author, year | Selection | | | | Comparability | | Outcome | | | Quality score |
| --- | --- | --- | --- | --- | --- | --- | --- | --- | --- | --- |
|  | Q1 | Q2 | Q3 | Q4 | Q5 | Q6 | Q7 | Q8 | Q9 |  |
| Damman et al., 2017 | X | √ | √ | √ | √ | √ | √ | √ | √ | 8 |
| Damman et al., 2016 | X | √ | √ | √ | √ | x | √ | √ | √ | 7 |
| Keen et al., 2010 | X | √ | √ | √ | x | x | √ | √ | √ | 6 |
| Haugen et al., 2017 | X | √ | √ | √ | √ | x | √ | √ | √ | 7 |
| Haugen et al., 2016a | X | √ | √ | √ | √ | √ | √ | √ | √ | 8 |
| Haugen et al., 2016c | X | √ | √ | √ | √ | √ | √ | √ | √ | 8 |
| Kortekaas et al., 2014 | X | √ | √ | √ | √ | √ | √ | √ | √ | 8 |
| Kortekaas et al., 2015a | X | √ | √ | √ | √ | √ | √ | √ | √ | 8 |
| Kortekaas et al., 2016 | X | √ | √ | √ | √ | √ | √ | √ | √ | 8 |
| Mancarella et al., 2015 | X | √ | √ | √ | √ | √ | √ | √ | √ | 8 |
| Mathiessen et al., 2016 | X | √ | √ | √ | √ | √ | √ | √ | √ | 8 |
| van Beest et al., 2019 | X | √ | √ | √ | √ | √ | √ | √ | √ | 8 |
| Median quality score |  |  |  |  |  |  |  |  |  | 8 |

Q1: Representativeness of the exposed cohort

Q2: Exposed/unexposed groups are drawn from the same source

Q3: Adequate ascertainment of exposure

Q4: Outcome of interest not visible at the start of the study

Q5: Age, sex and BMI controlled for in the analysis

Q6: Additional factors controlled for in the analysis

Q7: Assessment of outcomes done blinded to other results

Q8: Follow up time long enough for outcome to occur

Q9: All participants accounted for at follow up

***Supplementary Table S3b: Quality Assessment for Case-Control Studies***

| Author, Year | Selection | | | | Comparability | | Exposure | | | Quality score |
| --- | --- | --- | --- | --- | --- | --- | --- | --- | --- | --- |
|  | Q1 | Q2 | Q3 | Q4 | Q5 | Q6 | Q7 | Q8 | Q9 |  |
| Iagnocco et al., 2005 | √ | X | x | √ | x | X | √ | √ | √ | 5 |
| Keen et al., 2008c | √ | X | √ | √ | √ | X | √ | √ | x | 6 |
| Kortekaas et al., 2013 | √ | √ | √ | √ | √ | √ | √ | √ | x | 8 |
| Mancarella et al., 2010 | √ | X | √ | √ | x | √ | √ | √ | x | 6 |
| Wittoek et al., 2010 | √ | X | √ | √ | x | √ | √ | √ | √ | 7 |
| Wittoek et al., 2011 | √ | X | √ | √ | x | X | √ | √ | √ | 6 |
| Saltzherr et al., 2019 | √ | X | x | √ | x | X | √ | √ | √ | 5 |
| Median quality score |  |  |  |  |  |  |  |  |  | 6 |

Q1: Cases adequately and clearly defined

Q2: Representativeness of the targeted case population

Q3: Controls are drawn from the same population as cases

Q4: Controls clearly adequately and defined

Q5: Age, sex and BMI controlled for in analysis

Q6: Additional factors controlled for during analysis

Q7: Adequate ascertainment of exposure

Q8: Same method of ascertainment used for both cases and controls

Q9: Non-response rate same in both the cases and controls

***Supplementary Table S3c: Quality Assessment for Cross-Sectional Studies***

| Author, Year | Selection | | | | | Comparability | | Outcome | | | Quality score |
| --- | --- | --- | --- | --- | --- | --- | --- | --- | --- | --- | --- |
|  | Q1 | Q2 | Q3 | Q4 | Q5 | Q6 | Q7 | Q8 | Q9 | Q10 |  |
| Abraham et al., 2014 | √ | x | √ | √ | √ | N/A | N/A | √ | √ | √ | 7 |
| Dejaco et al., 2017 | X | x | x | √ | √ | N/A | N/A | √ | √ | √ | 5 |
| Grainger et al., 2007 | X | x | x | √ | √ | N/A | N/A | √ | √ | √ | 5 |
| Haugen et al., 2011 | X | x | x | √ | √ | N/A | N/A | √ | √ | √ | 5 |
| Hammer et al., 2016 | X | x | x | √ | √ | N/A | N/A | √ | √ | √ | 5 |
| Haugen et al., 2012a | X | x | √ | √ | √ | √ | X | √ | √ | √ | 7 |
| Haugen et al., 2012b | X | x | √ | √ | √ | √ | √ | √ | √ | √ | 8 |
| Haugen et al., 2015 | X | x | x | √ | √ | N/A | N/A | √ | √ | √ | 5 |
| Haugen et al., 2014 | X | x | x | √ | √ | N/A | N/A | √ | √ | √ | 5 |
| Keen et al., 2008a | X | x | x | √ | √ | N/A | N/A | √ | √ | √ | 5 |
| Jans et al., 2013 | X | x | x | √ | √ | N/A | N/A | √ | √ | √ | 5 |
| Iagnocco et al., 2012 | X | x | x | √ | √ | N/A | N/A | √ | √ | √ | 5 |
| Keen et al., 2008b | X | x | x | √ | x | N/A | N/A | √ | √ | √ | 4 |
| Kortekaas et al., 2011 | X | x | x | √ | √ | √ | √ | √ | √ | √ | 7 |
| Kortekaas et al., 2010 | X | x | x | √ | √ | √ | √ | √ | √ | √ | 7 |
| Kortekaas et al., 2015b | X | x | x | √ | √ | √ | X | √ | √ | √ | 6 |
| Kroon et al., 2018 | X | x | √ | √ | √ | √ | √ | √ | √ | √ | 8 |
| Kroon et al., 2017 | X | x | √ | √ | √ | X | X | √ | √ | √ | 6 |
| Liu et al., 2017 | X | x | √ | √ | √ | √ | √ | √ | √ | √ | 8 |
| Magnusson et al., 2017 | √ | x | x | √ | √ | √ | √ | √ | √ | √ | 8 |
| Mathiessen et al., 2013 | X | x | x | √ | √ | √ | X | √ | √ | √ | 6 |
| Paschoal et al., 2017 | X | x | x | √ | √ | X | X | √ | √ | √ | 5 |
| Ramonda et al., 2016 | X | x | x | √ | √ | X | X | √ | √ | √ | 5 |
| Sivakumaran et al., 2018 | X | x | x | √ | √ | X | X | √ | √ | √ | 5 |
| Uson et al., 2014 | X | x | x | √ | √ | N/A | N/A | √ | √ | √ | 5 |
| Vlychou et al., 2009 | X | x | x | √ | √ | N/A | N/A | √ | √ | √ | 5 |
| Fjellstad et al., 2019 | X | √ | x | √ | √ | √ | √ | √ | √ | √ | 8 |
| Kroon et al., 2019 | X | x | x | √ | √ | N/A | N/A | √ | √ | √ | 5 |
| Mattap et al., 2020 | √ | √ | √ | √ | x | √ | √ | √ | √ | √ | 9 |
| Oo et al., 2012 | √ | x | x | √ | √ | √ | √ | √ | √ | √ | 8 |
| Petterson et al., 2020 | X | √ | x | √ | √ | √ | √ | √ | √ | √ | 8 |
| van Beest et al., 2019 | X | x | x | √ | √ | √ | X | √ | √ | √ | 6 |
| Median quality score |  |  |  |  |  |  |  |  |  |  | 6 |

Q1: Representativeness of targeted population

Q2: Sample size justification

Q3: Summary of non-respondents recorded

Q4: Adequate ascertainment method used

Q5: Additional ascertainment method applied

Q6: Age, sex and BMI controlled in analysis

Q7: Additional factors controlled for in analysis

Q8: Assessment methods clearly defined

Q9: Assessment blinded to other results

Q10: Adequate statistical analysis

**Supplementary Table S4: Inter-rater reliability of detecting ultrasound features of hand osteoarthritis**

| Author, year | Sample size | No. of joints | Joints | Assessment type | Reliability measures | Reliability estimate (95% CI) | Definition | Scoring method | Probe frequency (MHz) |
| --- | --- | --- | --- | --- | --- | --- | --- | --- | --- |
|  |  |  | Osteophytes |  |  |  |  |  |  |
| Abraham, 2014 | 25 | 100 | 1st CMC | Static | Kappa | 0.69 (0.42, 0.95) | Keen, 2008 | Unclear | 10-18 |
|  |  |  | 2nd MCP |  |  | 0.5 (0.02, 0.99) |  |  |  |
|  |  |  | 2nd PIP |  |  | 0.62 (0.3, 0.93) |  |  |  |
|  |  |  | 2nd DIP |  |  | 0.69 (0.39, 1.0) |  |  |  |
| Wittoek, 2010 | 10 | 180 | IPJs | Static | Kappa | 0.98 | OMERACT | NA | 10-18 |
| Hammer, 2016 | 5 | 150 | CMC, MCP, PIP, DIP (semi-quantitative assessment) | Dynamic | Kappa mean | 0.67 (0.64, 0.69) |  | Mathiessen, 2013 Atlas | 6-15 |
|  |  |  | (Dichotomous assessment) |  |  | 0.58 (0.51, 0.63) |  |  |  |
| Keen, 2008b | 7 | 105 | CMC, MCP, PIP, DIP | Dynamic | Kappa | 0.53 | OMERACT | NA | 8-14 |
| Wittoek, 2011 | 5 | 90 | IPJs | Dynamic | Kappa | 0.83 (0.75, 0.91) | OMERACT | NA | 12-18 |
| Mathiessen, 2013 | 10 | 150 | CMC, MCP, PIP, DIP (session 1) | Static | Kappa weighted | 0.91 | OMERACT | Mathiessen, 2013 Atlas | 5-13 |
|  | 10 | 150 | CMC, MCP, PIP, DIP (session 2) | Static | Kappa weighted | 0.96 | OMERACT | Mathiessen, 2013 Atlas |  |
| Keen, 2008b | 7 | 105 | CMC, MCP, PIP, DIP | Dynamic | Kappa weighted | 0.378 | OMERACT | NA | 8-14 |
| Fjellstad, 2019 | 10 | 20 | CMC | Dynamic | Kappa weighted | 0.8 | Keen, 2008 | NA | 6-15 |
|  | 10 | 80 | IPJ | Dynamic | Kappa weighted | 0.72 | Keen, 2008 | NA |  |
|  |  |  | Cartilage abnormality |  |  |  |  |  |  |
| Hammer, 2016 |  | 125 | MCP (semi-quantitative assessment) | Static | Kappa mean | 0.47 (0.34, 0.56) |  |  |  |
|  |  |  |  |  | Grade 1 | 0.22 (0.16, 0.27) |  |  |  |
|  |  |  |  |  | Grade 2 | 0.3 (0.24, 0.36) |  |  |  |
|  |  |  |  |  | Grade 3 | 0.68 (0.58, 0.75) |  |  |  |
| Hammer, 2016 |  | 125 | MCP (dichotomous assessment) | Static | Kappa mean | 0.82 |  |  |  |
|  |  |  |  |  | Grade 1 | 0.61 |  |  |  |
|  |  |  |  |  | Grade 2 | 0.66 |  |  |  |
|  |  |  |  |  | Grade 3 | 0.87 |  |  |  |
| Hammer, 2016 | 5 | 40 | MCP (semi-quantitative assessment) | Dynamic | Kappa mean | 0.39 (0.31, 0.45) |  | Self-developed atlas | 8-18 |
|  |  |  | (dichotomous assessment) |  |  | 0.28 (0.15, 0.38) |  |  |  |
| Iagnocco, 2012 | 8 | 40 | MCP (dichotomous assessment) | Dynamic | Kappa mean | 0.62 | OMERACT | NA | 18 |
|  |  |  | Erosion |  |  |  |  |  |  |
| Wittoek, 2010 | 10 | 180 | IPJs | Static | Kappa | 0.91 | OMERACT | NA | 10-18 |
| Wittoek, 2011 | 5 | 90 | IPJs | Dynamic | Kappa | 0.9 (0.83, 0.97) | OMERACT | NA | 12-18 |
|  |  |  | Effusion |  |  |  |  |  |  |
| Wittoek, 2010 | 10 | 180 | IPJs | Static | Kappa | 0.98 | OMERACT | NA | 10-18 |
| Wittoek, 2011 | 5 | 90 | IPJs | Dynamic | Kappa | 0.84 (0.76, 0.92) | OMERACT | NA | 12-18 |
|  |  |  | Grey scale synovitis |  |  |  |  |  |  |
| Wittoek, 2010 | 10 | 180 | IPJ | Static | Kappa | 0.99 | OMERACT | NA | 10-18 |
| Keen, 2008b | 7 | 105 | CMC, MCP, PIP, DIP | Dynamic | Kappa | 0.398 | OMERACT | NA | 8-14 |
| Paschoal, 2017 | 15 | 15 | IPJ (scan on dorsal side) | Dynamic | Kappa | 0.617 | OMERACT | Szkudlarek, 2003 | 10-18 |
|  |  |  | (scan on palmar side) |  | Kappa | 0.498 | OMERACT | Szkudlarek, 2003 |  |
| Wittoek, 2011 | 5 | 90 | IPJ | Dynamic | Kappa | 0.93 (0.86, 1.0) | OMERACT | NA | 12-18 |
| Magnusson, 2017 | Not stated | Unclear | Unclear | Unclear | Kappa range | 0.75-0.94 | Keen, 2008 | Hammer, 2011 Atlas | Unclear |
| Mathiessen, 2016 | Unclear | 103 | CMC, MCP, PIP, DIP | Static | Kappa weighted | 0.74 | OMERACT | Unclear atlas | 5-13 |
| Keen, 2008b | 7 | 105 | CMC, MCP, PIP, DIP | Dynamic | Kappa weighted | 0.247 | OMERACT | NA | 8-14 |
| Fjellstad, 2019 | 10 | 20 | CMC | Dynamic | Kappa weighted | 0.92 | Keen, 2008 | NA | 6-15 |
|  | 10 | 80 | IPJ | Dynamic | Kappa weighted | 0.8 | Keen, 2008 | NA |  |
| Haugen, 2016 | 5 | 150 | CMC, MCP, PIP, DIP | Unclear | Kappa weighted | 0.63 | Hammer, 2011 Atlas | Hammer, 2011 Atlas | 5-13 |
| Pettersen, 2020 | 10 | 20 | PIP, DIP | Unclear | Kappa weighted | 0.8 | Keen, 2008 | Keen, 2008 | 6-15 |
| Paschoal, 2017 | 15 | 15 | IPJ (scan on dorsal side) | Dynamic | ICC | 0.494 | OMERACT | Szkudlarek, 2003 | 10-18 |
|  |  |  | IPJ (scan on palmar side) |  | ICC | 0.474 | OMERACT | Szkudlarek, 2003 |  |
|  |  |  | Power Doppler |  |  |  |  |  |  |
| Wittoek, 2010 | 10 | 180 | IPJs | Static | Kappa | 0.94 | OMERACT | NA | 8.3 |
| Keen, 2008b | 1 | 15 | CMC, MCP, PIP, DIP | Dynamic | Kappa | 0.327 | OMERACT | NA | 8-14 |
| Pettersen, 2020 | 10 | 20 | PIP, DIP | Unclear | Kappa | 0.79 | Keen, 2008 | NA | 7.7 |
| Mathiessen, 2016 |  | 20 | CMC, MCP, PIP, DIP | Static | Kappa weighted | >0.93 | OMERACT | Unclear atlas | 7.3 |
| Keen, 2008b | 1 | 15 | CMC, MCP, PIP, DIP | Dynamic | Kappa weighted | 0.229 | OMERACT | NA | 8-14 |
| Fjellstad, 2019 | 10 | 20 | CMC | Dynamic | Kappa weighted | 0.92 | Keen, 2008 | NA | 6-15 |
|  | 10 | 80 | IPJ | Dynamic | Kappa weighted | 0.85 | Keen, 2008 | NA |  |
| Haugen, 2016 | 5 | 150 | CMC, MCP, PIP, DIP | Unclear | Kappa weighted | 0.9 | Hammer, 2011 Atlas | Hammer, 2011 Atlas | 5-13 |

No.-number; CI-confidence interval, MHz-megahertz; CMC-carpometacarpal; MCP-metacarpophalangeal; PIP-proximal interphalangeal; DIP-distal interphalangeal; IPJ-interphalangeal joint; ICC-intra-class correlation coefficient; OMERACT-outcome measures in rheumatology; NA-not applicable

**Supplementary Table S5: Intra-rater reliability of detecting ultrasound features of hand osteoarthritis**

| Author | NP | NJ | Joints | Assessment type | Reliability Measures | Effect size | Definition | Scoring method | Time gap |
| --- | --- | --- | --- | --- | --- | --- | --- | --- | --- |
|  |  |  | Osteophytes |  |  |  |  |  |  |
| Hammer, 2016 | 5 | 150 | CMC, MCP, PIP, DIP | Dynamic | Kappa range (0-3) | 0.68 - 0.89  (0.75-0.84, 0.92-0.96) |  | Mathiessen, 2013 Atlas | 3 hours |
|  |  |  |  |  | (0-1) | 0.59 - 0.89  (0.43-0.72, 0.83-0.95) |  |  | 3 hours |
| Keen, 2008a | 10 | 240 | CMC, MCP, PIP, DIP | Dynamic | Kappa | 0.832 |  | Not stated | 4 weeks |
| Keen, 2008b | 1 | 15 | CMC, MCP, PIP, DIP | Dynamic | Kappa (Assessor1) | 0.375 | OMERACT | NA |  |
|  |  |  |  |  | Kappa (Assessor2) | 0.842 | OMERACT | NA |  |
|  |  |  |  |  | Kappa (Assessor3) | 0.087 | OMERACT | NA |  |
|  |  |  |  |  | Kappa (Assessor5) | 0.762 | OMERACT | NA |  |
|  |  |  |  |  | Kappa (Assessor6) | 1 | OMERACT | NA |  |
|  |  |  |  |  | Kappa (Assessor7) | 1 | OMERACT | NA |  |
| Keen, 2008c | 10 | 300 | CMC, MCP, PIP, DIP | Dynamic | Kappa | 0.83 | OMERACT | Keen, 2008 | 4 weeks |
| Vlychou, 2009 | 22 | 660 | CMC, MCP, PIP, DIP | Dynamic | Kappa | 0.81 | OMERACT | NA | 1 day |
| Sivakumaran, 2018 | U | U | CMC, MCP, PIP, DIP | Unclear | Kappa | 0.92 | OMERACT | Omeract | Unclear |
| Mathiessen, 2013 | 10 | 150 | CMC, MCP, PIP, DIP | Static | Kappa weighted  (assessor 1) | 0.95 | OMERACT | Mathiessen, 2013 Atlas | 1 week |
|  | 10 | 150 | CMC, MCP, PIP, DIP | Static | Kappa weighted  (assessor 2) | 0.98 | OMERACT | Mathiessen, 2013 Atlas | 1 week |
| Oo, 2019 | 40 | 40 | Thumb base | Static | Kappa weighted | 0.79 (0.63, 0.96) | OMERACT | Mathiessen, 2013 Atlas | 6 months |
| Keen, 2008b | 1 | 15 | CMC, MCP, PIP, DIP | Dynamic | Kappa weighted  (Assessor1) | 0.813 | OMERACT | NA |  |
|  |  |  |  |  | Kappa weighted  (Assessor2) | 0.935 | OMERACT | NA |  |
|  |  |  |  |  | Kappa weighted  (Assessor3) | 0.452 | OMERACT | NA |  |
|  |  |  |  |  | Kappa weighted  (Assessor4) | 0.763 | OMERACT | NA |  |
|  |  |  |  |  | Kappa weighted  (Assessor5) | 0.9 | OMERACT | NA |  |
|  |  |  |  |  | Kappa weighted  (Assessor6) | 0.973 | OMERACT | NA |  |
|  |  |  |  |  | Kappa weighted  (Assessor7) | 6.56 | OMERACT | NA |  |
| Mattap, 2019 | 20 | 300 | CMC, MCP, PIP, DIP | Dynamic | Kappa weighted | 0.753 (0.73, 0.76) | Keen, 2008 | Keen, 2008 | 1 day |
| Kortekaas, 2013 | 5 | 90 | IPJs | Dynamic | ICC | 0.71 | OMERACT | Szkudlarek | 5 hours |
| Kortekaas, 2015 | 6 | 48 | PIP, DIP | Unclear | ICC range | 0.62 - 0.91 |  |  |  |
| Kroon, 2018 |  |  |  |  | ICC | 0.62 - 0.91 |  |  |  |
| Uson, 2014 | 5 | 80 | PIP, DIP | Video clip | Percentage agreement | 98 | OMERACT | NA | 1 month |
|  |  |  | JSN |  |  |  |  |  |  |
| Hammer, 2016 | 5 | 40 | MCP | Static | Kappa range G0 | 0.87 - 1 |  | Self-developed atlas | 3 hours |
|  |  |  |  |  | Grade 1 cart lesion | 0.6 – 1 |  |  |  |
|  |  |  |  |  | Grade 2 cart lesion | 0.7 - 0.92 |  |  |  |
|  |  |  |  |  | Grade 3 cart lesion | 0.83 - 1 |  |  |  |
| Hammer, 2016 | 5 | 40 | MCP | Dynamic  (dichotomous) | Kappa range for cartilage score | 0.46 - 0.66  (0.35-0.68, 0.67-0.86) |  | Self-developed atlas | 3 hours |
|  |  |  |  | Semi-quantitative |  | -0.65 - 0.68  (-0.09-0.0, 0.31-0.92) |  |  | 3 hours |
| Keen, 2008a | 10 | 240 | CMC, MCP, PIP, DIP | Dynamic | Kappa | 0.641 | Not stated | Not stated | 4 weeks |
| Keen, 2008c | 10 | 300 | CMC, MCP, PIP, DIP | Dynamic | Kappa | 0.64 | OMERACT | Keen, 2009 | 4 weeks |
| Uson, 2014 | 5 | 80 | PIP, DIP | Video clip | Percentage agreement | 100% (for both JSN and cartilage absence) | OMERACT | NA | 1 month |
|  |  |  | Erosion |  |  |  |  |  |  |
| Mancarella, 2015 | 15 | 270 | PIP, DIP | Dynamic | Kappa | 0.87 (0.81, 0.93) | OMERACT |  | 1 week |
| Vlychou, 2009 | 22 | 660 | CMC, MCP, PIP, DIP | Dynamic | Kappa | 0.81 | OMERACT | NA | 1 day |
| Uson, 2014 | 5 | 80 | PIP, DIP | Video clip | Percentage agreement | 100% | OMERACT | NA | 1 month |
|  |  |  | Effusion |  |  |  |  |  |  |
| Wittoek, 2010 | 10 | 180 | IPJs | Static | Kappa | 0.98 | OMERACT | NA |  |
| Wittoek, 2011 | 5 | 90 | IPJs | Dynamic | Kappa | 0.84 (0.76, 0.92) | OMERACT | NA |  |
| Iagnocco, 2011 | 8 | 40 | MCP | Dynamic | Kappa | 0.54-0.94 | OMERACT | NA | 1 day |
|  |  |  | Grey scale synovitis |  |  |  |  |  |  |
| Keen, 2008b | 1 | 15 | CMC, MCP, PIP, DIP | Dynamic | Kappa (Assessor1) | 0.602 | OMERACT | NA | Not stated |
|  |  |  |  |  | Kappa (Assessor2) | 0.471 | OMERACT | NA | Not stated |
|  |  |  |  |  | Kappa (Assessor3) | 0.444 | OMERACT | NA | Not stated |
|  |  |  |  |  | Kappa (Assessor4) | 0.074 | OMERACT | NA | Not stated |
|  |  |  |  |  | Kappa (Assessor5) | 0.224 | OMERACT | NA | Not stated |
|  |  |  |  |  | Kappa (Assessor6) | 1 | OMERACT | NA | Not stated |
|  |  |  |  |  | Kappa (Assessor7) | 0.444 | OMERACT | NA | Not stated |
| Kortekaas, 2015 | 6 | 180 | CMC, MCP, PIP, DIP | Dynamic | Kappa | 0.73 | OMERACT | Szkudlarek | 5 hours |
| Mancarella, 2015 | 15 | 270 | PIP, DIP | Dynamic | Kappa | 0.84 (0.76, 0.93) | OMERACT |  | 1 week |
| Paschoal, 2017 | 15 | 15 | IPJ (scan on dorsal side) | Dynamic | Kappa | 0.673 | OMERACT | Szkudlarek, 2003 | 1, 4, 8, 12, 48 weeks |
|  |  |  | IPJ (scan on palmar side) |  | Kappa | -0.044 | OMERACT | Szkudlarek, 2003 | 1, 4, 8, 12, 48 weeks |
| Vlychou, 2009 | 22 | 660 | CMC, MCP, PIP, DIP | Dynamic | Kappa | 0.81 | OMERACT | NA | 1 day |
| Magnusson, 2017 | U | U | Unclear | Unclear | Kappa range | 0.75-0.94 | Keen, 2008 | Hammer, 2011 Atlas | Not stated |
| Mancarella, 2010 | 35 | 980 | MCP, PIP, DIP | Unclear | Kappa | 0.91 (0.843, 0.977) | OMERACT | NA | 12 weeks |
| Keen, 2008c | 10 | 300 | CMC, MCP, PIP, DIP | Static | Kappa weighted | 0.62 | OMERACT | Keen, 2010 | 12 weeks |
| Mathiessen, 2016 | U | 103 | CMC, MCP, PIP, DIP | Static | Kappa weighted | >0.86 | OMERACT | Unclear atlas | 1 week |
| Oo, 2019 | 40 | 40 | Thumb base | Static | Kappa weighted | 0.77 (0.6, 0.94) | Keen, 2008 | Keen, 2008 | 6 months |
| Keen, 2008b | 1 | 15 | CMC, MCP, PIP, DIP | Dynamic | Kappa weighted  (Assessor1) | 0.923 | OMERACT | NA | Not stated |
|  |  |  |  |  | Kappa weighted (Assessor2) | 0.673 | OMERACT | NA | Not stated |
|  |  |  |  |  | Kappa weighted  (Assessor3) | 0.637 | OMERACT | NA | Not stated |
|  |  |  |  |  | Kappa weighted  (Assessor4) | 0.753 | OMERACT | NA | Not stated |
|  |  |  |  |  | Kappa weighted  (Assessor5) | 0.545 | OMERACT | NA | Not stated |
|  |  |  |  |  | Kappa weighted  (Assessor6) | 1 | OMERACT | NA | Not stated |
|  |  |  |  |  | Kappa weighted  (Assessor7) | 0.369 | OMERACT | NA | Not stated |
| Mattap, 2019 | 20 | 300 | CMC, MCP, PIP, DIP | Dynamic | Kappa weighted | 0.661 (0.586, 0.719) | Keen, 2008 | Keen, 2008 | 1 day |
| Damman, 2016 | 6 | 180 | CMC, MCP, PIP, DIP | Dynamic | ICC | 0.93 | Keen, 2008 | Szkudlarek | 5 hours |
| Kortekaas, 2013 | 5 | 90 | IPJs | Dynamic | ICC | 0.73 | OMERACT | Szkudlarek | 5 hours |
| Kortekaas, 2016 | 5 | 90 | IPJs | Dynamic |  | 0.93 | OMERACT | Szkudlarek | 5 hours |
| Paschoal, 2017 | 15 | 15 | IPJ | Dynamic | ICC | 0.736 | OMERACT | Szkudlarek, 2003 | 1, 4, 8, 12, 48 weeks |
|  |  |  |  |  | ICC | 0.852 | OMERACT | Szkudlarek, 2003 | 1, 4, 8, 12, 48 weeks |
| Uson, 2014 | 5 | 80 | PIP, DIP | Video clip | Percentage agreement | 100 | OMERACT | NA | 1 month |
| Kroon, 2018 | 6 | 48 | PIP, DIP | Unclear | ICC range | 0.62 - 0.91 |  |  |  |
| Kortekaas, 2015 | 6 | 48 | PIP, DIP | Unclear | ICC range | 0.62 - 0.91 |  |  |  |
|  |  |  | Power Doppler |  |  |  |  |  |  |
| Oo, 2019 | 40 | 40 | Thumb base | Static | Kappa | 0.89 (0.69, 1.0) | OMERACT | NA | 6 months |
| Keen, 2008b | 1 | 15 | CMC, MCP, PIP, DIP | Dynamic | Kappa (Assessor1) | 0.471 | OMERACT | NA | Not stated |
|  |  |  |  |  | Kappa (Assessor2) | 0.667 | OMERACT | NA | Not stated |
|  |  |  |  |  | Kappa (Assessor3) | 0.571 | OMERACT | NA | Not stated |
|  |  |  |  |  | Kappa (Assessor4) | 0.211 | OMERACT | NA | Not stated |
|  |  |  |  |  | Kappa (Assessor5) | 0.595 | OMERACT | NA | Not stated |
|  |  |  |  |  | Kappa (Assessor6) | 1 | OMERACT | NA | Not stated |
|  |  |  |  |  | Kappa (Assessor7) | 0.25 | OMERACT | NA | Not stated |
| Kortekaas, 2015 | 6 | 180 | CMC, MCP, PIP, DIP | Dynamic | Kappa | 0.57 | OMERACT | Szkudlarek | 5 hours |
| Kortekaas, 2010 | 6 | 180 | CMC, MCP, PIP, DIP | Dynamic | Kappa | 0.57 | OMERACT | Szkudlarek | 5 hours |
| Mancarella, 2015 | 15 | 270 | PIP, DIP | Dynamic | Kappa | 0.78 (0.68, 0.89) | OMERACT |  | 1 week |
| Vlychou, 2009 | 22 | 660 | CMC, MCP, PIP, DIP | Dynamic | Kappa | 0.81 | OMERACT | NA | 1 day |
| Mancarella, 2010 | 35 | 980 | MCP, PIP, DIP | Unclear | Kappa | 0.864 (0.748, 0.979) | OMERACT | NA | 12 weeks |
| Keen, 2008c | 10 | 300 | CMC, MCP, PIP, DIP | Static | Kappa weighted | 0.87 | OMERACT | Keen, 2011 | 12 weeks |
| Mathiessen, 2016 | U | 20 | CMC, MCP, PIP, DIP | Static | Kappa weighted | >0.86 | OMERACT | Unclear atlas | 1 week |
| Keen, 2008b | 1 | 15 | CMC, MCP, PIP, DIP | Dynamic | Kappa weighted  (Assessor1) | 0.305 | OMERACT | NA | Not stated |
|  |  |  |  |  | Kappa weighted  (Assessor2) | 0.83 | OMERACT | NA | Not stated |
|  |  |  |  |  | Kappa weighted  (Assessor3) | 0.439 | OMERACT | NA | Not stated |
|  |  |  |  |  | Kappa weighted  (Assessor4) | 0.276 | OMERACT | NA | Not stated |
|  |  |  |  |  | Kappa weighted  (Assessor5) | 0.637 | OMERACT | NA | Not stated |
|  |  |  |  |  | Kappa weighted  (Assessor6) | 1 | OMERACT | NA | Not stated |
|  |  |  |  |  | Kappa weighted  (Assessor7) | 0.277 | OMERACT | NA | Not stated |
| Mattap, 2019 | 20 | 300 | CMC, MCP, PIP, DIP | Dynamic | Kappa weighted | 0.689 (0.525, 0.78) | Keen, 2008 | Keen, 2008 | 1 day |
| Damman, 2016 | 6 | 180 | CMC, MCP, PIP, DIP | Dynamic | ICC | 0.62 |  | Szkudlarek | 5 hours |
| Kortekaas, 2013 | 5 | 90 | IPJs | Dynamic | ICC | 0.57 | OMERACT | Szkudlarek | 5 hours |
| Kortekaas, 2011 | 6 | 180 | CMC, MCP, PIP, DIP | Dynamic | ICC | 0.57 | OMERACT | Szkudlarek | 5 hours |
| Kortekaas, 2016 | 5 | 90 | IPJs | Dynamic | ICC | 0.62 | OMERACT | Szkudlarek | 5 hours |
| Kroon, 2018 |  |  |  |  | ICC | 0.62 - 0.91 |  |  |  |
| Kortekaas, 2015 | 6 | 48 | PIP, DIP | Unclear | ICC range | 0.62 - 0.91 |  |  |  |

No.-number; CI-confidence interval, MHz-megahertz; CMC-carpometacarpal; MCP-metacarpophalangeal; PIP-proximal interphalangeal; DIP-distal interphalangeal; IPJ-interphalangeal joint; ICC-intra-class correlation coefficient; OMERACT-outcome measures in rheumatology; NA-not applicable

**Supplementary Table S6: Inter-rater reliability of detecting MRI features of hand osteoarthritis**

| **Author, year** | **NP** | **NJ** | **Joints** | **Reliability Measures** | **Point estimate (95% CI)** | **Definition** | **Scoring method** |
| --- | --- | --- | --- | --- | --- | --- | --- |
|  |  |  |  | **Osteophytes** |  |  |  |
| Haugen, 2011 | 10 | 80 | PIP, DIP | Average measure ICC | 0.91 (0.58, 0.98) | Oslo HOA score | Oslo HOA score |
|  |  |  |  | Single measure ICC (median from the 3 assessors) | 0.88 (0.86, 0.89) | Oslo HOA score | Oslo HOA score |
| Haugen, 2015 | 20 | 160 | PIP, DIP | Average measure ICC (baseline assessment) | 0.74 (0.19, 0.91) | OMERACT HOA | OMERACT HOA score |
|  | 20 | 160 | PIP, DIP | Average measure ICC (follow up assessment) | 0.76 (0.13, 0.92) | OMERACT HOA | OMERACT HOA score |
| Haugen, 2014 | 8 | 64 | PIP, DIP | Average measure ICC (1^st^ session) | 0.88 (0.68, 0.97) | Oslo HOA score | Oslo HOA score |
|  | 8 | 64 | PIP, DIP | Average measure ICC (2^nd^ session) | 0.97 (0.9, 0.99) | OMERACT HOA | OMERACT HOA score |
| Kroon, 2017 | 20 | 20 | Thumb | ICC average measure | 0.83 (0.56, 0.93) | OMERACT TOMS | OMERACT TOMS score |
| Ramonda, 2016 | 11 | 88 | PIP, DIP | ICC single measure  (distal joint side) | 0.48 (0.27, 0.65) | Oslo HOA | Oslo HOA score |
|  |  |  |  | ICC single measure  (proximal joint side) | 0.48 (0.26, 0.65) | Oslo HOA | Oslo HOA score |
| Kroon, 2018 | 25 | 25 | CMC | ICC average measure for change of score | 0.47 (-0.02, 0.75) | OMERACT TOMS | OMERACT TOMS |
| van Beest, 2019 | 10 | 10 | CMC and STT | ICC average measure range reported for all features | 0.72-0.92 | OMERACT TOMS | OMERACT TOMS |
| Jans, 2013 | 9 | 72 | PIP, DIP | Kappa | 0.12 | OMERACT definitions, Ostergaard, 2006 |  |
| Wittoek, 2011 | 14 | 112 | IPJs | Kappa | 0.15 (0.04, 0.27) | OMERACT HOA | NA |
|  |  |  |  | **Joint space narrowing** |  |  |  |
| Haugen, 2011 | 10 | 80 | PIP, DIP | Average measure ICC | 0.99 (0.95, 1.0) | Oslo HOA score | Oslo HOA |
|  |  |  |  | Single measure ICC (median from the 3 assessors) | 0.97 (0.93, 0.99) | Oslo HOA score | Oslo HOA |
| Haugen, 2015 | 20 | 160 | PIP, DIP | Average measure ICC (baseline cartilage space assessment) | 0.93 (0.72, 0.98) | OMERACT HOA | OMERACT HOA score |
|  | 20 | 160 | PIP, DIP | Average measure ICC (follow up cartilage space assessment) | 0.92 (0.71, 0.97) | OMERACT HOA | OMERACT HOA score |
| Haugen, 2014 | 8 | 64 | PIP, DIP | Average measure ICC (1^st^ session for cartilage space loss) | 0.88 (0.65, 0.97) | Oslo HOA score | Oslo HOA score |
|  | 8 | 64 | PIP, DIP | Average measure ICC (2^nd^ session for cartilage space loss) | 0.98 (0.93, 0.99) | OMERACT HOA | OMERACT HOA score |
| Kroon, 2017 | 20 | 20 | Thumb | ICC average measure (cartilage abnormality) | 0.79 (0.48, 0.92) | OMERACT TOMS | OMERACT TOMS score |
| Ramonda, 2016 | 11 | 88 | PIP, DIP | ICC single measure | 0.59 (0.4, 0.73) | Oslo HOA score | Oslo HOA score |
| Kroon, 2018 | 25 | 25 | CMC | ICC average measure for change of score (cartilage abnormality) | 0.39 (-0.18, 0.71) | OMERACT TOMS | OMERACT TOMS |
|  | 25 | 25 | STT | ICC average measure for change of score (cartilage abnormality) | 0.72 (0.47, 0.87) | OMERACT TOMS | OMERACT TOMS |
| van Beest, 2019 | 10 | 10 | CMC and STT | ICC average measure range reported for all features | 0.72-0.92 | OMERACT TOMS | OMERACT TOMS |
| Saltzherr, 2019 | 10 | 233 | 2 and 3 MCP and PIP | Kappa weighted (for JSN) | 0.39 (0.22, 0.54) | OMERACT HOA | OMERACT score |
|  |  | 234 |  | Kappa weighted (for cartilage score) | 0.63 (0.54, 0.7) | OMERACT HOA | OMERACT score |
|  |  |  |  | **Erosion** |  |  |  |
| Haugen, 2011 | 10 | 80 | PIP, DIP | Average measure ICC | 0.94 (0.74, 0.99) | Oslo HOA score | Oslo HOA score |
| Haugen, 2015 | 20 | 160 | PIP, DIP | Average measure ICC (Baseline assessment) | 0.81 (0.32, 0.93) | OMERACT HOA | OMERACT HOA score |
|  | 20 | 160 | PIP, DIP | Average measure ICC (follow up assessment) | 0.84 (0.33, 0.95) | OMERACT HOA | OMERACT HOA score |
| Haugen, 2014 | 8 | 64 | PIP, DIP | Average measure ICC (1^st^ session) | 0.83 (0.57, 0.96) | Oslo HOA score | Oslo HOA score |
|  | 8 | 64 | PIP, DIP | Average measure ICC (2^nd^ session) | 0.96 (0.9, 0.99) | OMERACT HOA | OMERACT HOA score |
| Kroon, 2018 | 20 | 20 | Thumb | ICC average measure (SBD) | 0.88 (0.73, 0.95) | OMERACT TOMS | OMERACT TOMS score |
| Ramonda, 2016 | 11 | 88 | PIP, DIP | ICC single measure (distal) | 0.51 (0.3, 0.67) | Oslo HOA score | Oslo HOA score |
|  |  |  |  | ICC single measure (proximal) | -0.31 (-0.07, 0.52) | Oslo HOA score | Oslo HOA score |
| Kroon, 2018 | 25 | 25 | CMC | ICC average measure for change of score (SBD) | 0.72 (0.47, 0.87) | OMERACT TOMS | OMERACT TOMS |
|  | 25 | 25 | STT | ICC average measure for change of score (SBD) | 0.71 (0.44, 0.86) | OMERACT TOMS | OMERACT TOMS |
| van Beest, 2019 | 10 | 10 | CMC and STT | ICC average measure range reported for all features | 0.72-0.92 | OMERACT TOMS | OMERACT TOMS |
| Grainger, 2007 | 15 | 15 | 1 DIP or PIP | Kappa | 0.84 | OMERACT (Ostergaard, 2003) | OMERACT (Ostergaard, 2003) |
| Jans, 2013 | 9 | 72 | PIP, DIP | Kappa | 0.7 | OMERACT (Ostergaard, 2003) |  |
| Wittoek, 2011 | 14 | 112 | IPJs | Kappa | 0.76 (0.64, 0.88) | OMERACT HOA | NA |
|  |  |  |  | **Bone marrow lesions** |  |  |  |
| Haugen, 2011 | 10 | 80 | PIP, DIP | Average measure ICC | 0.83 (0.51, 0.96) | Oslo HOA score | Oslo HOA score |
|  |  |  |  | Single measure ICC (median from the 3 assessors) | 0.89 (0.65, 0.89) | Oslo HOA score | Oslo HOA score |
| Haugen, 2015 | 20 | 160 | PIP, DIP | Average measure ICC (Baseline) | 0.87 (0.72, 0.94) | OMERACT HOA | OMERACT HOA score |
|  | 20 | 160 | PIP, DIP | Average measure ICC (follow up) | 0.91 (0.79, 0.97) | OMERACT HOA | OMERACT HOA score |
| Haugen, 2014 | 8 | 64 | PIP, DIP | Average measure ICC (1^st^ session) | 0.94 (0.84, 0.99) | Oslo HOA score | Oslo HOA score |
|  | 8 | 64 | PIP, DIP | Average measure ICC (2^nd^ session) | 0.79 (0.39, 0.95) | OMERACT HOA | OMERACT HOA score |
| Kroon, 2017 | 20 | 20 | Thumb | ICC average measure | 0.99 (0.98, 1.0) | OMERACT TOMS | OMERACT TOMS score |
| Ramonda, 2016 | 11 | 88 | PIP, DIP | ICC single measure (distal) | 0.34 (0.1, 0.53) | Oslo HOA score | Oslo HOA score |
|  |  |  |  | ICC single measure (proximal) | 0.34 (0.1, 0.54) | Oslo HOA score | Oslo HOA score |
| Kroon, 2018 | 25 | 25 | CMC (session 1 including all rheumatology trained) | ICC average measure for change of score | 0.84 (0.69, 0.93) | OMERACT TOMS | OMERACT TOMS |
|  | 25 | 25 | STT | ICC average measure for change of score | 0.92 (0.83, 0.96) | OMERACT TOMS | OMERACT TOMS |
|  | 24 | 24 | CMC (session 2 including a radiologist) | ICC average measure for change of score | 0.89 (0.75, 0.95) | OMERACT TOMS | OMERACT TOMS |
|  | 16 | 16 | STT | ICC average measure for change of score | 0.9 (0.68, 0.97) | OMERACT TOMS | OMERACT TOMS |
| Jans, 2013 | 9 | 72 | PIP, DIP | Kappa | 0.77 | OMERACT (Ostergaard, 2004) |  |
|  |  |  |  | **Effusion** |  |  |  |
|  |  |  |  |  |  |  |  |
| Wittoek, 2011 | 14 | 112 | IPJs | Kappa | 0.5 (0.35, 0.65) | OMERACT HOA | NA |
|  |  |  |  | **Synovitis** |  |  |  |
| Haugen, 2011 | 10 | 80 | PIP, DIP | Average measure ICC | 0.84 (0.5, 0.96) | Oslo HOA | Oslo HOA score |
|  |  |  |  | Single measure ICC (median from the 3 assessors) | 0.48 (0.09, 0.7) |  |  |
| Haugen, 2015 | 20 | 160 | PIP, DIP | Average measure ICC (baseline) | 0.5 (0.05, 0.78) | OMERACT HOA | OMERACT HOA score |
|  | 20 | 160 | PIP, DIP | Average measure ICC (follow up) | 0.57 (0.09, 0.82) | OMERACT HOA | OMERACT HOA score |
| Haugen, 2014 | 8 | 64 | PIP, DIP | Average measure ICC (1^st^ session) | 0.34 (-0.14, 0.79) | Oslo HOA | Oslo HOA score |
|  | 8 | 64 | PIP, DIP | Average measure ICC (2^nd^ session) | 0.74 (0.3, 0.94) | OMERACT HOA | OMERACT HOA score |
| Kroon, 2017 | 20 | 20 | Thumb | ICC average measure | 0.81 (0.6, 0.92) | OMERACT TOMS | OMERACT TOMS score |
| Ramonda, 2016 | 11 | 88 | PIP, DIP | ICC single measure | 0.41 (0.16, 0.6) | Oslo HOA | Oslo HOA score |
| Kroon, 2018 | 25 | 25 | CMC (session 1 all rheumatology trained) | ICC average measure for change of score | 0.83 (0.68, 0.92) | OMERACT TOMS | OMERACT TOMS |
|  | 25 | 25 | STT | ICC average measure for change of score | 0.56 (0.12, 0.79) | OMERACT TOMS | OMERACT TOMS |
|  | 24 | 24 | CMC (session 2 including a radiologist) | ICC average measure for change of score | 0.55 (-0.07, 0.8) | OMERACT TOMS | OMERACT TOMS |
| Jans, 2013 | 9 | 72 | PIP, DIP | Kappa | 0.77 | OMERACT (Ostergaard, 2005) |  |
| Wittoek, 2011 | 14 | 112 | IPJs | Kappa | 0.58 (0.4, 0.76) | OMERACT HOA | NA |

No.-number; CI-confidence interval, MHz-megahertz; CMC-carpometacarpal; MCP-metacarpophalangeal; PIP-proximal interphalangeal; DIP-distal interphalangeal; IPJ-interphalangeal joint; ICC-intra-class correlation coefficient; OMERACT-outcome measures in rheumatology; NA-not applicable; OMREACT TOMS-thumb base osteoarthritis MRI scoring system; Oslo HOA- Oslo hand osteoarthritis MRI scoring method; T1W-T1 weighted; T2W-T2 weighted; FSE-fast spin echo, SE-spin echo; Fat Sat-fat saturation

**Supplementary Table S7: Intra-rater reliability of detecting MRI features of hand osteoarthritis**

| **Author, year** | **NP** | **NJ** | **Joints** | **Reliability measures** | **Reliability estimate (95% CI)** | **Definition** | **Scoring method** | **Time gap** |
| --- | --- | --- | --- | --- | --- | --- | --- | --- |
|  |  |  |  | **Osteophytes** |  |  |  |  |
| Haugen, 2017 | 7 | 48 | PIP, DIP | Kappa median (IQR) osteophyte proliferation | 0.7 (0.36, 1.0) |  | OMERACT HOA score | 2 weeks |
| Haugen, 2012a | 10 | 80 | PIP, DIP | Kappa weighted | 0.71 | Oslo HOA | Oslo HOA score | 7 weeks |
| Haugen, 2012b | 10 | 80 | PIP, DIP | ICC | 0.95 | Oslo HOA | Oslo HOA score | 7 weeks |
| Kortekaas, 2015 | 6 | 48 | PIP, DIP | ICC (Distal) | 0.92 | Oslo HOA | Oslo HOA score | 5 weeks |
|  |  |  |  | ICC (proximal) | 0.86 | Oslo HOA | Oslo HOA score | 5 weeks |
| Kroon, 2017 | 20 | 20 | Thumb | ICC single measure range reported CMC | 0.71-0.73 | OMERACT TOMS | OMERACT TOMS score | 1 month |
|  |  |  |  | STT | 0.44-0.71 | OMERACT TOMS | OMERACT TOMS score | 1 month |
| Ramonda, 2016 | 11 | 88 | PIP, DIP | ICC average measure distal | 0.66 (0.43, 0.79) | Oslo HOA | Oslo HOA score | Unclear |
|  |  |  |  | ICC average measure proximal | 0.61 (0.35, 0.76) | Oslo HOA | Oslo HOA score | Unclear |
| van Beest, 2019 | 16 | 128 | PIP, DIP | ICC | 0.9 | OMERACT | OMERACT score | Unclear |
| van Beest, 2019 | 10 | 10 | CMC and STT | ICC single measure range reported for all features | 0.76-1.0 | OMERACT TOMS | OMERACT TOMS | Unclear |
|  |  |  |  | **Joint space narrowing** |  |  |  |  |
| Haugen, 2012 | 10 | 80 | PIP, DIP | Kappa weighted | 0.77 | Oslo HOA | Oslo HOA score | 7 weeks |
| Saltzherr, 2019 | 10 | 233 | 2 and 3 MCP and PIP | Kappa weighted (JSN) | 0.59 (0.38, 0.79) | OMERACT | OMERACT score | 4 months |
|  |  | 234 |  | Kappa weighted (cartilage score) | 0.62 (0.15, 0.8) | OMERACT | OMERACT score | 4 months |
|  |  |  |  | ICC | 0.93 | Oslo HOA | Oslo HOA score | 7 weeks |
| Kortekaas, 2015 | 6 | 48 | PIP, DIP | ICC | 0.88 | Oslo HOA | Oslo HOA score | 5 weeks |
| Kroon, 2017 | 20 | 20 | Thumb-CMC | ICC single measure range reported | 0.61-0.86 | OMERACT TOMS | OMERACT TOMS score | 1 month |
|  |  |  | STT |  | 0.71-0.84 | OMERACT TOMS | OMERACT TOMS score | 1 month |
| Ramonda, 2016 | 11 | 88 | PIP, DIP | ICC average measure | 0.88 (0.79, 0.92) | Oslo HOA | Oslo HOA score | Unclear |
| van Beest, 2019 | 10 | 10 | CMC and STT | ICC single measure range reported for all features | 0.76-1.0 | OMERACT TOMS | OMERACT TOMS | Unclear |
|  |  |  |  | **Erosion** |  |  |  |  |
| Haugen, 2017 | 7 | 48 | PIP, DIP | Kappa median (IQR) | 0.72 (0.62, 1.0) |  | OMERACT HOA score | 3 weeks |
| Haugen, 2012 | 10 | 80 | PIP, DIP | Kappa weighted | 0.84 | Oslo HOA | Oslo HOA score | 7 weeks |
|  |  |  |  | ICC | 0.92 | Oslo HOA | Oslo HOA score | 7 weeks |
| Kortekaas, 2015 | 6 | 48 | PIP, DIP | ICC (distal) | 0.91 | Oslo HOA | Oslo HOA score | 5 weeks |
|  |  |  |  | (proximal) | 0.87 | Oslo HOA | Oslo HOA score | 5 weeks |
| Kroon, 2017 | 20 | 20 | Thumb | ICC single measure range reported SBD CMC | 0.89 - 0.89 | OMERACT TOMS | OMERACT TOMS score | 1 month |
|  |  |  |  | SBD STT | 0.62 - 0.7 | OMERACT TOMS | OMERACT TOMS score | 1 month |
| Ramonda, 2016 | 11 | 88 | PIP, DIP | ICC average measure (distal) | 0.59 (0.33, 0.75) | Oslo HOA | Oslo HOA score | Unclear |
|  |  |  |  | ICC average measure (proximal) | 0.66 (0.44, 0.79) | Oslo HOA | Oslo HOA score | Unclear |
| van Beest, 2016 | 10 | 10 | CMC and STT | ICC single measure range reported for all features | 0.76-1.0 | OMERACT TOMS | OMERACT TOMS | Unclear |
|  |  |  |  | **Bone marrow lesions** |  |  |  |  |
| Haugen, 2016 | 5 | 40 | PIP, DIP | Kappa for change of score | 0.76 | OMERACT HOA | OMERACT HOA score | NA |
| Haugen, 2012 | 10 | 80 | PIP, DIP | Kappa weighted | 0.77 | Oslo HOA | Oslo HOA score | 7 weeks |
| Damman, 2017 | 11 | 88 | PIP, DIP | ICC | 0.84 - 1.0 | Nieuwenhuis, 2015 | Modified Oslo scoring | Not stated |
| Haugen, 2012 | 10 | 80 | PIP, DIP | ICC | 0.94 | Oslo HOA | Oslo HOA score | 7 weeks |
| Kortekaas, 2015 | 6 | 48 | PIP, DIP | ICC (distal) | 0.89 | Oslo HOA | Oslo HOA score | 5 weeks |
|  |  |  |  | (proximal) | 0.87 | Oslo HOA | Oslo HOA score | 5 weeks |
| Kroon, 2018 | Un | Un | CMC | ICC | 0.92 | OMERACT TOMS | OMERACT TOMS score | Not stated |
|  |  |  | STT | ICC | 0.91 | OMERACT TOMS | OMERACT TOMS score | Not stated |
| Kroon, 2017 | 20 | 20 | CMC | ICC single measure range reported | 0.96-0.98 | OMERACT TOMS | OMERACT TOMS score | 1 month |
|  |  |  | STT |  | 0.87-0.92 | OMERACT TOMS | OMERACT TOMS score | 1 month |
| Liu, 2017 | 11 | 88 | PIP, DIP | ICC | >0.97 | Oslo HOA | Modified Oslo HOA score | 3 weeks |
| Ramonda, 2016 | 11 | 88 | PIP, DIP | ICC average measure (distal) | 0.51 (0.18, 0.7) | Oslo HOA | Oslo HOA score | Unclear |
|  |  |  |  | ICC average measure (proximal) | 0.56 (0.28, 0.73) | Oslo HOA | Oslo HOA score | Unclear |
| van Beest, 2019 | 16 | 128 | PIP, DIP | ICC | 0.91 | OMERACT | OMERACT score | Unclear |
|  |  |  |  | **Effusion** |  |  |  |  |
| Damman, 2017 | 11 | 88 | PIP, DIP | ICC | 0.84 - 1.0 | Nieuwenhuis, 2015 | Modified Oslo scoring | Not stated |
|  |  |  |  | **Synovitis** |  |  |  |  |
| Haugen, 2016 | 5 | 40 | PIP, DIP | Kappa change of score | 0.77 | OMERACT HOA | OMERACT HOA score | NA |
| Haugen, 2012a | 10 | 80 | PIP, DIP | Kappa weighted | 0.78 | Oslo HOA | Oslo HOA score | 7 weeks |
| Haugen, 2016 | 5 | 40 | PIP, DIP | Kappa weighted | 0.76 | OMERACT HOA | OMERACT HOA score | Unclear |
| Haugen, 2012b | 10 | 80 | PIP, DIP | ICC | 0.91 | Oslo HOA | Oslo HOA score | 7 weeks |
| Kortekaas, 2015 | 6 | 48 | PIP, DIP | ICC | 0.94 | Oslo HOA | Oslo HOA score | 5 weeks |
| Kroon, 2018 | Un | Un | CMC | ICC | 0.65 | OMERACT TOMS | OMERACT TOMS score | Not stated |
|  |  |  | STT | ICC | 0.58 | OMERACT TOMS | OMERACT TOMS score | Not stated |
| Kroon, 2017 | 20 | 20 | CMC | ICC single measure range reported | 0.53 - 0.83 | OMERACT TOMS | OMERACT TOMS score | 1 month |
|  |  |  | STT |  | 0.72 - 0.89 | OMERACT TOMS | OMERACT TOMS score | 1 month |
| Liu, 2017 | 11 | 88 | PIP, DIP | ICC | >0.97 | Oslo HOA | Modified Oslo HOA score | 3 weeks |
| Ramonda, 2016 | 11 | 88 | PIP, DIP | ICC average measure | 0.63 (0.38, 0.79) | Oslo HOA | Oslo HOA score | Unclear |
| van Beest, 2019 | 16 | 128 | PIP, DIP | ICC | 0.91 | OMERACT | OMERACT score | Unclear |

No.-number; NP-sample size; NJ-number of joints; CI-confidence interval, MHz-megahertz; CMC-carpometacarpal; MCP-metacarpophalangeal; PIP-proximal interphalangeal; DIP-distal interphalangeal; IPJ-interphalangeal joint; ICC-intra-class correlation coefficient; OMERACT-outcome measures in rheumatology; NA-not applicable; OMREACT TOMS-thumb base osteoarthritis MRI scoring system; Oslo HOA- Oslo hand osteoarthritis MRI scoring method; T1W-T1 weighted; T2W-T2 weighted; FSE-fast spin echo, SE-spin echo; Fat Sat-fat saturation

**Supplementary Table S8: Subgroup analysis for ultrasound features based on training background of assessors, assessment type and probe frequency**

| **Ultrasound feature** | **Reliability measure** | **Assessors' training background**  **Reliability estimate (95% CI) [I^2^], number of studies** | | **Assessment type**  **Reliability estimate (95% CI) [I^2^], number of studies** | | **Probe frequency**  **Reliability estimate (95% CI) [I^2^], number of studies** | |
| --- | --- | --- | --- | --- | --- | --- | --- |
|  |  | Rheumatology | Non-Rheumatology | Dynamic | Static | >15 MHz | <15 MHz |
|  | **Inter-rater** |  |  |  |  |  |  |
| Osteophyte | Pooled Kappa | 0.70 (0.46, 0.95) [95.83%], n=2 | 0.61 (0.51, 0.71) [0.00%], n=2 | 0.67 (0.50, 0.83) [91.35%], n=3 | - | 0.69 (0.53, 0.84) [88.92%], n=3 |  |
|  | Pooled Kappa weighted | 0.77 (0.63, 0.92) [98.14%], n=3 | - | 0.61 (0.43, 0.80) [93.08%], n=3 | - | 0.69 (0.63, 0.75) [48.73%], n=2 | 0.66 (0.16, 1.16) [97.38], n=2 |
| Synovitis | Pooled Kappa | 0.77 (0.42, 1.12) [84.91%], n=2 | - | 0.64 (0.32, 0.97) [92.43%], n=3 | - | 0.77 (0.42, 1.12) [84.91], n=2 |  |
|  | Pooled Kappa weighted | 0.76 (0.61, 0.90) [74.15], n=3 | - | 0.56 (-0.04, 1.16) [96.76%], n=2 | - | 0.84 (0.74, 0.94) [0.01%], n=2 | 0.45 (0.07, 0.82) [92.48], n=2 |
| Power Doppler | Pooled Kappa | 0.90 (0.76, 1.03) [57.77%], n=2 | - | 0.68 (0.09, 1.27) [84.67%], n=2 | - |  |  |
|  | Pooled Kappa weighted | 0.90 (0.86, 0.93) [0.00%], n=2 | - | 0.60 (-0.03, 1.23) [84.95%], n=2 | - |  |  |
|  | **Intra-rater** |  |  |  |  |  |  |
| Osteophyte | Pooled Kappa | 0.83 (0.79, 0.87) [0.00%], n=4 | 0.81 (0.78, 0.83) [0.00%], n=2 | 0.81 (0.79, 0.84) [0.01%], n=4 | - | 0.84 (0.80, 0.88) [0.00%], n=2 | 0.67 (0.44, 0.89) [81.42%] |
|  | Pooled Kappa weighted | 0.88 (0.80, 0.96) [96.20%], n=3 | 0.87 (0.74, 1.01) [97.45%], n=2 | 0.84 (0.75, 0.93) [90.70%], n=2 | 0.96 (0.92, 0.99) [89.28%], n=2 |  | 0.94 (0.90, 0.97) [82.08], n=3 |
| Synovitis | Pooled ICC | 0.83 (0.71, 0.96) [85.23%], n=3 |  | 0.83 (0.71, 0.96) [85.23], n=3 | - |  |  |
|  | Pooled Kappa | 0.64 (0.47, 0.80) [88.64%], n=9 | 0.57 (0.01, 1.14) [81.38%], n=2 | 0.60 (0.44, 0.76) [90.98%], n=5 | - |  |  |
|  | Pooled Kappa weighted | 0.75 (0.65, 0.86) [79.59%], n=5 | 0.66 (0.59, 0.72) [0.00%], n=2 | 0.71 (0.58, 0.84) [68.73%], n=2 | 0.75 (0.60, 0.90) [90.41%], n=3 | 0.64 (0.59, 0.69) [0.02%], n=2 | 0.79 (0.69, 0.88) [58.11%], n=3 |
| Power Doppler | Pooled Kappa | 0.67 (0.53, 0.80) [76.25%], n=5 | 0.78 (0.64, 0.92) [0.01%], n=2 | 0.63 (0.51, 0.76) [78.05%], n=4 | - |  |  |
|  | Pooled Kappa weighted | 0.64 (0.42, 0.85) [86.81%], n=3 | 0.68 (0.56, 0.80) [0.00%], n=2 | 0.58 (0.41, 0.75) [53.99%], n=2 | 0.87 (0.84, 0.90) [0.01%], n=2 |  |  |
| Effusion | Pooled Kappa | 0.84 (0.71, 0.96) [90.59%], n=3 | - | 0.79 (0.74, 0.85) [61.94%], n=3 |  |  |  |

**Supplementary Table S9: Sensitivity analysis excluding studies that repeated real-time ultrasound scans on different days**

| **Ultrasound feature** | **Reliability measure** | **Excluding studies that did not acquire images on the same day**  **Reliability estimate (95% CI) [I^2^], number of studies** |
| --- | --- | --- |
|  |  |  |
|  | **Intra-rater** |  |
| Grey-scale Synovitis | Pooled Kappa weighted | 0.55 (0.38, 0.72) [87.99%], n=4 |
| Power Doppler | Pooled Kappa weighted | 0.64 (0.50, 0.78) [79.96%], n=4 |
| Effusion | Pooled Kappa | 0.78 (0.70, 0.85) [77.53%], n=2 |

**Supplementary Table S10: Sensitivity analysis excluding non-contrast enhanced MRI studies**

|  | Inter-rater reliability: ICC (95% CI) [I^2^], number of studies |
| --- | --- |
| Synovitis | 0.64 (0.47, 0.82) [61.53], n=5 |
| Bone marrow lesion | 0.81 (0.63, 0.99) [95.43], n=5 |
|  | Intra-rater reliability: ICC (95% CI) [I^2^], number of studies |
| Synovitis | 0.93 (0.89, 0.96) [79.25%], n=5 |
| Bone marrow lesion | 0.87 (0.74, 0.99) [98.75%] n=5 |

ICC-intra-class correlation coefficient; CI-confidence interval, I^2^-heterogeneity
